# Targeted Sequencing of Lung Function Loci in Chronic Obstructive Pulmonary Disease Cases and Controls

**DOI:** 10.1371/journal.pone.0170222

**Published:** 2017-01-23

**Authors:** María Soler Artigas, Louise V. Wain, Nick Shrine, Tricia M. McKeever, UK BiLEVE, Ian Sayers, Ian P. Hall, Martin D. Tobin

**Affiliations:** 1 Genetic Epidemiology Group, Department of Health Sciences, University of Leicester, Leicester, United Kingdom; 2 National Institute for Health Research (NIHR), Leicester Respiratory Biomedical Research Unit, Glenfield Hospital, Leicester, United Kingdom; 3 Division of Respiratory Medicine, Queen’s Medical Centre, University of Nottingham, Nottingham, United Kingdom; Hospital Authority, CHINA

## Abstract

Chronic obstructive pulmonary disease (COPD) is the third leading cause of death worldwide; smoking is the main risk factor for COPD, but genetic factors are also relevant contributors. Genome-wide association studies (GWAS) of the lung function measures used in the diagnosis of COPD have identified a number of loci, however association signals are often broad and collectively these loci only explain a small proportion of the heritability. In order to examine the association with COPD risk of genetic variants down to low allele frequencies, to aid fine-mapping of association signals and to explain more of the missing heritability, we undertook a targeted sequencing study in 300 COPD cases and 300 smoking controls for 26 loci previously reported to be associated with lung function. We used a pooled sequencing approach, with 12 pools of 25 individuals each, enabling high depth (30x) coverage per sample to be achieved. This pooled design maximised sample size and therefore power, but led to challenges during variant-calling since sequencing error rates and minor allele frequencies for rare variants can be very similar. For this reason we employed a rigorous quality control pipeline for variant detection which included the use of 3 independent calling algorithms. In order to avoid false positive associations we also developed tests to detect variants with potential batch effects and removed them before undertaking association testing. We tested for the effects of single variants and the combined effect of rare variants within a locus. We followed up the top signals with data available (only 67% of collapsing methods signals) in 4,249 COPD cases and 11,916 smoking controls from UK Biobank. We provide suggestive evidence for the combined effect of rare variants on COPD risk in *TNXB* and in sliding windows within *MECOM* and upstream of *HHIP*. These findings can lead to an improved understanding of the molecular pathways involved in the development of COPD.

## Introduction

Chronic obstructive pulmonary disease (COPD) is the third cause of death worldwide [1]. Forced expiratory volume in one second (FEV_1_) and the ratio of FEV_1_ to forced vital capacity (FVC), measured by spirometry, are used in the diagnosis of COPD. Whilst environmental factors such as air pollution or tobacco smoking have a negative effect on lung function and increase the risk of developing COPD [2, 3], both lung function measures and COPD are heritable [4–6]. Genome-wide association studies (GWAS) of FEV_1_ and FEV_1_/FVC have now collectively identified 44 loci that have an effect on lung function [7–12]. This study focuses on the 26 loci that were first identified. Out of these 26 loci, 12 have already shown association with COPD risk, either in GWAS of COPD risk [13–16] or in studies that have only analysed previously-reported lung function associated variants [17, 18]. The known risk variants overall tend to have small effect sizes and only explain a small proportion of the heritability [9]. Most genome-wide association studies of lung function undertaken to date have focused on identifying common variants (minor allele frequency, MAF, >5%), and it is hypothesized that variants with lower allele frequency might have larger effect sizes and therefore might play an important role in explaining the missing heritability [19]. A recent GWAS of lung function [12] using data imputed to the 1000 Genomes Project reference panel [20] identified two low frequency variants with larger effect sizes. In addition, many of the association signals for the loci known to affect lung function are not well localized, and identifying rare (MAF ≤ 1%) or low frequency (1% < MAF ≤ 5%) variants within these regions can aid the identification of the causal variants.

In order to detect genetic associations with COPD risk for variants down to low allele frequencies in 26 loci associated with lung function, we undertook a two-stage study. In stage 1 we sequenced these 26 loci in 300 COPD cases and 300 smoking controls, using a cost-effective pooled design to maximize the sample size. We undertook single variant analysis and applied two collapsing methods in order to increase the power to detect rare variant associations in these regions. In stage 2 we followed up the top signals in 4,249 COPD cases and 11,916 smoking controls within the UK BiLEVE study (a subset of UK Biobank) [11], with genotypes imputed to the joint 1000 Genomes Project [20] and UK10K [21] reference panel.

## Results

### Study design, sequencing and data processing

Three hundred COPD cases, defined using spirometry (GOLD stage 2 [3] and above), and 300 controls were selected among individuals over 40 years of age who were smokers from three studies: Gedling, Nottingham Smokers and the Leicester COPD cases. Spirometry procedures carried out in these studies can be found in [9, 22] and characteristics of individuals included in the study are presented in S1 Table.

The region sequenced for each of the 26 loci associated with FEV_1_ or FEV_1_/FVC was defined using the results from the largest meta-analysis of GWAS for these traits undertaken to date [9]. The parameters used in this definition were: distance from the most significantly associated SNP (sentinel SNP) in each region and strength of association, measured by P-value, (details in the Methods section and in S2 Table). In total 7.7Mb of sequence was covered and the sequencing was undertaken using Illumina HiSeq 2000 with 100 bp paired-end reads and 8 lanes, each with 3 pools. Each pool contained DNA from 25 cases or 25 controls. The data were aligned against 1000 Genomes Project phase 1 data [20] (GRCh37; h19) using BWA.6.2 [23]. Alignments were then sorted and PCR duplicates were removed using SAMtools [24] and indels were locally realigned and quality scores recalibrated using GATK v2.5 [25]. After removing two control pools (50 control individuals) due to lower DNA quality than the rest, the average coverage per individual was 30x.

### Variant calling and quality control checks

In order to distinguish true calls from sequencing error, three different calling algorithms specific for pooled data were used (vipR [26], SNVer [27] and Syzygy [28]). vipR was less sensitive than the other two algorithms and it called a much smaller number of variants, 39,211 SNPs and 459 deletions, compared to 62,506 SNPs and 5,811 indels by SNVer and 55,886 SNPs and 5,331 indels by Syzygy. Most of the variants called by vipR had MAF > 1% (85% of SNPs and 99% of indels). SNVer also called mainly variants with MAF > 1% (81% of SNPs and all indels), whereas Syzygy called the largest number of rare variants (43% SNPs and 35% of indels). In terms of specificity, Syzygy and vipR showed the best specificity when assessing the proportion of SNPs with MAF > 1% included in dbSNP137 [29] (97.08% for vipR and 98.2% for Syzygy), compared to a 72% for SNVer. When calling indels all algorithms had lower specificity than when calling SNPs, assessed as the proportion of indels with MAF > 1% included in 1000 Genomes Project phase 1 [20], (67.55% for vipR, 35% for SNVer and 50% for Syzygy).

**Fig 1:**
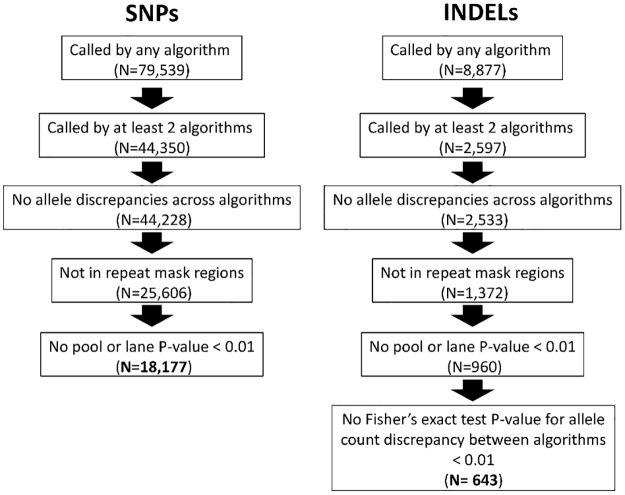
Flow chart of the variant selection process.

A series of quality control checks and filtering strategies were performed (Fig 1, details in the Methods section). More than 90% of SNPs (> 46% of indels) with MAF > 1% called by at least two algorithms were present in dbSNP137 [29], in contrast to < 70% of SNPs (< 40% for indels) with MAF > 1% called by only one algorithm (S1 Fig). This indicated that the set of variants called by at least two algorithms was more reliable, and therefore variants called only by one algorithm were excluded. Variants were also excluded if the alleles called by different algorithms did not agree or if they were in a repeat masked region. Additional tests were performed in order to remove variants which could generate false associations due to a lane or pool effect (see details in the Methods section). Allele frequencies for SNPs were consistent across algorithms and with those from 1000 Genomes Project phase 1 data [20] (S2a and S2b Fig). There were considerable discrepancies across algorithms for some indels, therefore, a Fisher’s exact test (FET) was performed for indel allele counts in order to exclude those that showed marked discrepancies across algorithms (S2c and S2d Fig). After undertaking quality control checks a total of 18,177 SNPs and 643 indels across the 26 regions were selected for association testing with COPD risk (Fig 1). Some of these variants were novel: 1,429 SNPs (93% with MAF < 1%) were not in dbSNP137 [29] or in the joint 1000 Genomes Project [20] and UK10K [21] reference panel and 216 indels (4% with MAF < 1%) were not in 1000 Genomes Project phase 1 data [20] or reported by Mills *et al*. [30]. There was a notable difference in the quality of the data for SNPs and indels. Out of all variants called by at least one algorithm, after all quality control checks 23% of SNPs remained, whereas for indels it was only a 7%.

### Single variant

Given the pooled design of the experiment, no allele counts per individual were available, only allele counts within sets of cases and controls; therefore the association testing was undertaken using Fisher’s exact test. It was performed separately for the allele counts produced by each calling algorithm, for the variants that passed the quality control checks. A total of 8 SNPs and 3 indels, different from the sentinel variants associated with lung function in previous studies [7–10], met a significance threshold adjusted for multiple testing (significance thresholds defined separately for each region, see Methods section and S3 Table) using the allele counts for at least one calling algorithm and showed supportive evidence (P < threshold x 2) when using allele counts from another calling algorithm (S4a Table).

These variants were followed up with data imputed to the joint 1000 Genomes Project [20] and UK10K [21] reference panel in 4,249 COPD cases and 11,916 smoking controls from the UK BiLEVE study [11] (characteristics in S1 Table). Only one variant (rs999741 in the *HTR4* region, MAF 26%) had a nominally significant P-value (P = 2x10^-3^) and had consistent direction of effect in stage 1 and stage 2 (S4 Table). When conditioned on the previously reported variant (rs1985524 [9]) in that region, rs999741 was no longer significant (S5 Table) thereby confirming that this signal was not independent of rs1985524 and confirming the previously reported association with COPD for this SNP.

Association results for the 26 previously reported lung function sentinel SNPs (or SNPs in perfect LD with the sentinel SNPs) are presented in S6 Table. Sentinel lung function SNPs in four regions (*MECOM*, *HHIP*, *SPATA9*, *HTR4*) reached nominally significant P-values (Table 1) when using allele counts for at least two calling algorithms and their direction of effect agreed with the previously reported [9] effect on lung function (negative effect on FEV_1_ or FEV_1_/FVC and increased risk of COPD, or positive effect on FEV_1_ or FEV_1_/FVC and reduced risk of COPD). Association with COPD risk for *HHIP* and *HTR4* had already been reported [15, 18], but not for *SPATA9* or *MECOM*. These four variants were also analysed in UK BiLEVE, where again the association of *HHIP* and *HTR4* were confirmed, but no association was found for *SPATA9* or *MECOM* (Table 1).

**Table 1:** COPD association results in stage 1 and 2 for lung function sentinel variants analysed in stage 2.

rs number	GWAS gene	Ref allele	Alt allele	Calling algorithm	Stage 1				Stage 2	
					Alt allele freq in cases	Alt allele freq in controls	OR	P-value	OR	P-value
rs1344555	*MECOM*	C	T	SNVer	0.238	0.154	1.72	4.93x10^−4^	1.03	4.20x10^−1^
				Syzygy	0.247	0.162	1.69	5.93x10^−4^		
				vipR	0.260	0.180	1.6	9.29x10^−3^		
rs11100860	*HHIP*	A	G	SNVer	0.337	0.410	0.73	1.44x10^−2^	0.86	3.31x10^−9^
				Syzygy	0.340	0.404	0.76	3.28x10^−2^		
				vipR	0.398	0.406	0.97	8.37x10^−1^		
rs153916	*SPATA9*	C	T	SNVer	0.600	0.530	1.33	2.03x10^−2^	0.99	5.55x10^−1^
				Syzygy	0.605	0.536	1.33	2.35x10^−2^		
				vipR	0.576	0.526	1.09	1.33x10^−1^		
rs1985524	*HTR4*	G	C	SNVer	0.378	0.448	0.75	2.27x10^−2^	0.88	1.08x10^−6^
				Syzygy	0.383	0.446	0.77	3.67x10^−2^		
				vipR	0.428	0.446	0.93	5.89x10^−1^		

COPD single variant results for lung function sentinel variants [9] with P < 0.05 when using allele counts for at least two calling algorithms in stage 1 and 2. Full stage 1 results for the 26 lung function loci are presented in S6 Table. OR correspond to the alternative allele.

Abbreviations: Ref = reference, Alt = alternative, freq = frequency, N = number, ac = allele count, OR = odds ratio.

### Collapsing methods

In order to increase power to identify associations with rare variants we applied two collapsing methods. A burden test was applied using Fisher’s exact test to assess whether accumulation of rare variants in a locus (number of individuals with at least one rare allele) was associated with COPD risk. The C-alpha test [31] was also applied to test whether a locus was associated with COPD risk allowing for variants to be protective or detrimental. Only rare SNPs (MAF < 1%) were included in these analyses and loci boundaries were defined in three different ways: (i) sliding window: 3kb sliding windows with an overlap of 1.5kb, (ii) gene based: gene coordinates, and (iii) exon based: exons, 5’ UTR and 3’ UTR for each gene.

A total of 59 3kb sliding windows from 18 regions out of the 26 sequenced, and 23 genes (21 from gene based tests, 1 from exon based tests and 1 that was selected for both) from 19 regions out of the 26 sequenced met a significance threshold adjusted for multiple testing (see Methods section and S3 Table) using the allele counts for at least one calling algorithm and showed supportive evidence (P < threshold x 2) when using allele counts from another calling algorithm (S7 Table). Of these, two sliding windows and three genes from the gene based analysis were selected due to their P-values in the burden analysis; all the remaining windows and genes were selected because of their C-alpha test P-values. Only enough variants were available to follow-up 32 sliding windows out of the 59 and the 23 genes in UK BiLEVE. Full stage 2 results are provided in S8 Table.

None of the two sliding windows and three genes selected for their burden test P-values reached nominal significance (P<0.05) in UK BILEVE (S8a Table). The C-alpha test results for sliding windows showed one sliding window in the *MECOM* region (chr3:169238286–169241286) which met a threshold corrected for multiple testing for that region (P < 8x10^-3^), and sliding windows in the *RARB* region (chr3:25633833–25636833) and in the *HHIP* region (chr4:145293600–145296600) that showed suggestive evidence of association (P = 0.05 and P = 0.04 respectively) (Table 2). For the C-alpha test for gene based analysis, *C10orf11* met the significance threshold for that region (P < 0.05, only one gene in the region) and *TNXB*, in the AGER region, showed near-suggestive evidence of association (P = 0.06) (Table 2). For the C-alpha test for exon based analysis, *NPNT* in the *GSTCD* region showed suggestive evidence of association (P = 0.05, Table 2). Full results for the C-alpha test are presented in S8b Table.

**Table 2:** Collapsing methods results for most significant loci in stage 2.

Locus, (GWAS gene)	Stage 1		Stage 2							
	Threshold	P-value	Threshold	All variants			Independent variants			
				Number of variants	Number of alternative allele counts	P-value	Number of variants	Number of alternative allele counts	P-value	P-value after permutations
chr3:25633833–25636833, *(RARB)*	5.81x10^−4^	9.43x10^−7^	1.25x10^−2^	3	363	5.32x10^−2^	3	363	5.32x10^−2^	6.25x10^−2^
chr3:169238286–169241286, *(MECOM)*	1.87x10^−4^	1.46x10^−5^	8x10^−3^	2	573	**2.94x10**^**−3**^	2	573	**2.94x10**^**−3**^	1.92x10^−2^
chr4:145293600–145296600, *(HHIP)*	2.76x10^−4^	1.11x10^−5^	1.25x10^−2^	2	167	4.16x10^−2^	2	167	4.16x10^−2^	6.19x10^−2^
*NPNT* (chr4:106816596–106892828), (*GSTCD)*	1.25x10^−2^	1.81x10^−3^	2.5x10^−2^	9	1400	5.25x10^−2^	3	422	5.1x10^−1^	4.07x10^−1^
*TNXB* (chr6:32008931–32077151), *(AGER)*	7.14x10^−3^	6.91x10^−3^	5x10^−2^	37	8086	6.08x10^−2^	11	1752	**4.73x10**^**−2**^	6.69x10^−2^
*C10orf11* (chr10:77542518–78317126) *(C10orf11)*	5x10^−2^	3.95x10^−18^	5x10^−2^	304	39634	**4.03x10**^**−2**^	124	15529	**2.77x10**^**−2**^	5x10^−1^

“GWAS gene” presents the gene previously reported for lung function [9] for each of the 26 regions. The most significant stage 1 P-values across the three algorithms for the analysis only including independent variants are presented here; for full results see S7 Table. Stage 2 P-values that meet the threshold are shown in bold.

After repeating the analyses for these six loci keeping only independent variants (r^2^ < 0.2, see Methods) in each locus, as a sensitivity analysis, *NPNT* was no longer significant, *TNXB* and *C10orf11* met the significance threshold corrected for multiple testing, and the sliding window results remained the same. The C-alpha test authors (53) recommend undertaking permutations for the top loci. Ten thousand permutations were run for the 6 most significant loci including only independent variants. After permutations, *NPNT* and *C10orf11* were not significant and suggestive evidence was provided for the remaining loci (P = 6.25x10^-2^ for chr3:25633833–25636833 in *RARB*, P = 1.92x10^-2^ for chr3:169238286–169241286 in *MECOM*, P = 6.19x10^-2^ chr4:145293600–145296600 upstream of *HHIP* and P = 6.69x10^-2^ *TNXB*).

In order to gain more insights into the 4 loci that showed suggestive evidence of association, single variant results for the variants included in each region were examined and the C-alpha test was undertaken again removing one variant at a time to assess the single variant effect on the results both in stage 1 and stage 2 (S3 Fig). The signal in the sliding window in *RARB* was driven by the same variant in stage 1 and in stage 2 however, the direction of effect did not agree between stages, indicating that this was probably a false positive association. The remaining signals seemed to be driven by different variants in stage 1 and stage 2.

## Discussion

The aim of this study was to identify low frequency and rare variants in genetic regions known to be associated with lung function in order to gain insights into the biological pathways that link these regions with COPD risk. To do this, 26 regions associated with lung function [7–10] were sequenced in 300 COPD cases and 300 controls using a cost-effective pooled design. In order to minimize the occurrence of false positive calls three variant calling algorithms were used in this study. Single and multi (collapsing) variant association analyses were undertaken and the strongest signals were followed up in 4,249 COPD cases and 11,916 controls from the UK BiLEVE study [11]. Suggestive evidence of association with COPD risk was shown for a window in *MECOM*, one intergenic window upstream of *HHIP* and for the *TNXB* gene in the *AGER* region.

The strongest collapsing signal in stage 2 was for a sliding window (chr3:169238286–169241286) in an intronic region of *MECOM*, which includes a DNase hypersensitivity site for blood microvascular endothelial cells derived from lung tissue [32]. Variants in *MECOM* (r^2^ <0.03 with rs1344555) have been associated with osteoporosis [33], renal function-related traits [34] and nasopharyngeal carcinoma [35] in East Asians and with blood pressure [36] and magnesium levels [37] in Europeans. The MDS1 and EVI1 complex locus protein (MECOM) encodes a number of transcripts that code for nuclear transcription factors [38]. Overexpression of the oncoprotein ecotropic virus integration site 1 protein homolog (EVI1) has been associated with multiple epithelial cancers, such as nasopharyngeal carcinoma, lung and colorectal cancers [35, 39–41]. EVI1 is also involved in embryonic development, through a role in haematopoiesis and mouse knock out models for MECOM have shown embryonic lethality [42–44].

The sliding window ~270kb upstream of *HHIP* that showed suggestive evidence of association with COPD is located in a region that contains a DNase hypersensitivity site and transcription factors binding sites found in blood cells, renal epithelium cells and embryonic stem cells [32]. This region does not overlap with another region ~85kb upstream of *HHIP* known to interact with the *HHIP* promoter and to function as an *HHIP* enhancer [45].

Single variant association analyses for sentinel variants previously associated with lung function [9] confirmed the previously reported associations with COPD risk for *HHIP* [15] and *HTR4* [18].

Given the pooled nature of the stage 1 design it was not possible to adjust the phenotype for any covariates. Both cases and controls were individuals of over 40 years of age, who smoked between 5 and 100 pack years; and COPD case control status was determined using FEV_1_ percent predicted, which takes into account age, sex and height. Analyses in stage 2 were undertaken as close as possible to stage 1, and therefore the selection criterion was the same as in stage 1.

A limitation of this study was its reduced sample size. Assuming a COPD prevalence of 30% among smokers, a study with 300 cases and 300 controls would need an OR of 5 in order to detect a variant with MAF ~ 1% and an OR of 2 for a variant with MAF ~ 5% with 80% power at a nominal level of significance (P = 0.05). However, this study was designed to identify genetic associations with rare variants, which are expected to have larger effect sizes [19]. We decided to use a pooled sequencing design in order to maximise stage 1 sample size, however this design also has limitations. It is challenging to determine whether a single variant is homozygous or heterozygous, particularly if the allele frequency of this variant is rare, limiting this way the ability of this approach to discover new variants reliably. In addition, there are a number of factors that can affect the quality of the sequencing data, such as PCR amplification biases, reference allele preferential biases or varying error sequencing rates across sites [46]. This led us to apply three different calling algorithms using different assumptions and statistical models and to apply strict filters. In some instances these filters might have been over conservative.

The three calling algorithms used to minimise the number of false calls used different statistical methods and they also performed differently. vipR and SNVer called mainly variants with MAF > 1%, vipR being the least sensitive of the three algorithms. Syzygy called the largest number of rare variants and along with vipR were the algorithms with best specificity. Calling of indels was more challenging than calling SNPs regardless of which algorithm was used.

The key strength of this study was the ability to identify novel low frequency or rare variants through sequencing. A rigorous analytic pipeline was followed in this study overall. Support from at least two of the three calling algorithms was required for a variant to be called; strict filters were also applied assessing for example potential pool or lane effects. In addition, in order to select the most robust associations with COPD risk, results for at least one calling algorithm had to meet a Bonferroni threshold and support was also required from results using another calling algorithm. Moreover, in order to distinguish real associations from spurious ones, replication was pursued in an independent study. This study despite providing data only for 67% of collapsing methods signals, most of which were imputed rather than genotyped, had a considerably larger sample size and its power was enhanced by the sampling strategy used, selecting individuals from extremes of the lung function distribution in UK Biobank.

Overall, this pooled sequencing study, which implemented strict filtering strategies, identified 18,177 SNPs and 643 indels. It showed suggestive evidence for the association of rare variants with COPD risk in sliding windows in *MECOM* and upstream of *HHIP* and in *TNXB*. These findings will contribute to improve the knowledge of the biological mechanisms underlying the COPD and may lead to the development of new preventive and treatment strategies.

## Methods

### Samples in stage 1

Individuals from three studies were included in this analysis: Gedling, Nottingham Smokers and the Leicester COPD cases. Spirometry procedures for Gedling and Nottingham Smokers can be found in [9] and for the Leicester COPD cases in [22]. Individuals were excluded if (i) they were younger than 40 years old, (ii) they had pack-years of smoking < 5, or > 100, or (iii) if they had DNA concentration ≤ 20ng/uL. Individuals with asthma were also excluded from the Leicester COPD cases study. This left a sampling frame of 965 individuals (403 from Gedling, 468 from Nottingham Smokers and 96 from the Leicester COPD cases). COPD cases were defined as spirometric GOLD stage 2 [3] and above (percent predicted FEV_1_ < 80% and FEV_1_/FVC < 0.7) and controls as individuals with percent predicted FEV_1_ > 80% and FEV_1_/FVC > 0.7, based on pre-bronchodilator spirometry. Individuals with percent predicted FEV_1_ > 80% and FEV_1_/FVC < 0.7 (GOLD stage 1 [3]) or with percent predicted FEV_1_ < 80% and FEV_1_/FVC > 0.7 were excluded from the analysis to minimize misclassification. The calculation of percent predicted FEV_1_ was undertaken using reference values of FEV_1_ that take into account age, sex and height according to previously described equations [47, 48]. In order to select the most extreme 300 COPD cases and 300 smoking controls, COPD cases and smoking controls were ranked according to their percent predicted FEV_1_ and selected from the extremes. In order to remove extremely healthy individuals from the controls, individuals were excluded if (i) they had percent predicted FEV_1_ > 120.26 (the 99th percentile of percent predicted FEV_1_) or (ii) if they had FEV_1_/FVC > 0.85 (the 95th percentile of FEV_1_/FVC). Individuals were grouped into pools of 25 (separately for cases and controls), following the percent predicted FEV_1_ ranking, so that individuals with more similar phenotype would be grouped together.

### Definition of regions

Region plots produced with data from the largest GWAS to date for FEV_1_ and FEV_1_/FVC measures [9] for the 26 loci associated with lung function [7–10] were examined to define the association regions. SNPs with −log10 (P-value) > 2.5 and not further than 50kb away from the next SNP with −log10 (P-value) > 2.5 moving away from the sentinel SNP, were selected. Any gene intersecting the association region was added to the region +/-10kb. If the association region did not include or intersect the closest gene, the association region was enlarged to include the closest gene +/- 10kb. If the enlarged regions also intersected other genes, the regions were not enlarged again, so they included small portions of genes. Regions were selected using the −*log*_10_ (*P* − *value*) for the most significant trait only, except for *CDC123* which was genome-wide significant for FEV_1_ and FEV_1_/FVC and the sentinel SNP was the same for both traits. For *CDC123* the association region was defined so it included the association regions for both traits. The regions covered a total of 10.3Mb (S2 Table).

### Sequencing method

The enrichment kit was produced by Agilent (http://www.agilent.com/) and the sequencing was undertaken by Source BioScience (http://www.sourcebioscience.com/). After applying a correction for GC content and applying repeat-masking filters, a total of 7.7Mb of sequence was covered by probes in the final design. Sequencing was undertaken using Illumina HiSeq2000 with 100 bp paired-end reads and 8 lanes, each with 3 pools. Pools were assigned to lanes sequentially, so that pool1 to pool3 were allocated to lane 1, pool4 to pool6 were allocated to lane 2 and so on; in total there were four case lanes and four control lanes.

### Alignment and data processing

The data were aligned against 1000 Genomes Project phase 1 reference panel [20] (GRCh37; h19) using BWA.6.2 [23], with −q 15 for read trimming. Alignments were then sorted and PCR duplicates were removed using SAMtools [24]. After the removal of duplicates, coverage summaries were produced using SAMtools [24] and BEDtools [49]. GATK [25] was used for base quality scores recalibration and local realignment around indels using known indel coordinates from 1000 Genomes Project phase 1 data [20] and Mills *et al*. data [30] as reference.

### Variant calling and quality control checks

In order to distinguish true calls from sequencing error, three different calling algorithms specific for pooled data were used (vipR [26], SNVer [27] and Syzygy [28]) and a series of quality control checks and filtering strategies were performed. Variants were excluded if they were called only by one calling algorithm, if the alleles called by different algorithms differed or if they were in repeat masked regions (extracted from UCSC table browser [50]). A lane test was designed to detect variants affected by lane effects. Given that lanes included only case pools or only control pools, a sequencing artefact in a lane could lead to a false association. A chi-square test with three degrees of freedom was run for the four case lanes and the four control lanes for each variant. A pool test was implemented to detect sequencing artefacts in pools which could lead to false associations and to assess whether the data were consistent within case pools and within control pools. A Binomial test was performed for each pool, to test whether the observed allele count would be expected given the number of chromosomes in the pool and the allele frequency observed across case or control pools (allele frequency was calculated separately for case pools and control pools). Variants with either a P-value < 0.01 in the lane test for case pools or control pools, or a P-value < 0.01 in the pool test for any pool were excluded. Allele frequencies obtained using allele counts from the different calling algorithms were compared across algorithms and with 1000 Genomes Project phase 1 data [20]. A Fisher’s exact test was performed for indel allele counts in order to exclude those that showed discrepancies across algorithms.

### Association testing

Variants selected were tested for association with COPD risk using Fisher’s exact tests on allele counts per pool (since no allele accounts per individual were available) using allele counts produced by the different calling algorithms both for SNPs and indels.

Collapsing method tests were only applied to SNPs (with MAF < 1%), due to the lower quality shown for data on indels. Loci boundaries were defined in three different ways, in order to detect associations in three different biological scenarios: (i) sliding window: 3kb sliding windows with an overlap of 1.5kb, (ii) gene based: gene coordinates, and (iii) exon based: exons, 5’ UTR and 3’ UTR for each gene. Gene, UTR and exon coordinates were extracted from UCSC table browser [50] using the RefSeq Genes track. A burden test based on [51] was undertaken using Fisher’s exact test to assess whether the accumulation of rare variants in a locus (number of individuals with at least one rare allele) was associated with COPD risk. In order to infer how many individuals had at least one rare allele it was assumed that individuals with the alternative allele would always be heterozygous (rather than homozygous, since only overall allele count per pool was available). In addition, it was assumed that rare variants within a locus were independent. In order to test whether a locus was associated with COPD risk allowing for variants to be protective or detrimental, the C-alpha test [31] was also applied. This test also assumes that variants within a region are independent. All association testing analyses were performed using R v3.1.0 (https://www.r-project.org/).

In order to assess the effect on the results of the assumption that variants with MAF < 1% in a locus were independent, the collapsing tests were run again for the most significant loci after removing variants in LD (r^2^ > 0.2) with each other. Within a group of variants in LD the one with the smallest P-value across the three calling algorithms was chosen. LD was calculated using the joint 1000 Genomes Project [20] and UK10K reference panel. The effect on the results of assuming that variants not present in this joint reference panel were independent or were in LD with any of the other variables in the region was assessed by running the collapsing methods with and without variants not in the joint 1000 Genomes Project [20] and UK10K [21] reference panel.

### Significance thresholds

Significance thresholds to account for multiple testing were defined for each of the 26 regions separately. As there is already strong prior evidence for the association of the 26 regions with lung function [7–10], no multiple testing adjustment for the number of regions was undertaken. For the single variant analysis the effective number of independent variants tested (equivalent to the number of independent tests) was estimated using the approach developed by Li and Ji [52], and then a Bonferroni correction for the number of independent tests was applied in each region. Data from the joint 1000 Genomes Project [20] and UK10K [21] reference panel were used to estimate the correlation matrix for each region. Variants not included in this reference panel were assumed to be independent. For the collapsing methods a Bonferroni correction was applied for the number of independent tests within each region. For the sliding windows, given the overlap between windows the number of independent tests was defined as half the number of sliding windows, and for the gene based and exon based, the number of independent tests was defined as the number of genes included in each analysis. Significance thresholds for each region and each test are provided in S3 Table.

### Selection of signals for follow-up

In order to minimise false positive associations, the criteria to select the top hits required that a variant met the significance threshold using allele counts for one calling algorithm and that it also showed supporting evidence (P-value < threshold x 2) when using allele counts from another calling algorithm. For the collapsing methods, loci had to meet this criterion also after the sensitivity analysis in order to be followed-up. Alignments were visually inspected for all the single variants selected and for a random sample of the variants in the collapsing method selected loci.

### Follow-up (stage 2)

Variants and loci selected were followed-up in UK BiLEVE [11], a subset of ~50,000 individuals from UK Biobank (http://www.ukbiobank.ac.uk/) sampled from the extremes of the % predicted FEV_1_ distribution separately in never smokers and heavy smokers. An Affymetrix Axiom custom array was designed for the genome-wide genotyping of the UK BiLEVE project, including 130K rare missense and loss of function variants, and 642K variants selected for optimal imputation of common variation and improved imputation of low frequency variation (MAF 1–5%), and 9000 variants selected for improved coverage of known and candidate respiratory regions. These data were imputed against the joint 1000 Genomes Project phase 1 [20] and UK10K [21] reference panel.

The sampling frame was made of 20,859 individuals over 40 years old, with no asthma (diagnosed or self-reported) who smoked between 5 and 100 pack years. Case-control status was defined as in the COPD case-control sequencing study. COPD cases defined as in stage 1, but percent predicted FEV_1_ was obtained using reference values from healthy (no respiratory diseases diagnosed) never smokers (N = 81,719) from UK Biobank. In total there were 4,249 COPD cases and 11,916 smoking controls.

The association of single variants with COPD risk in UK BiLEVE was tested using logistic regression on allele dosages obtained from the imputation output. An adjustment for 5 principal components of ancestry was included. All variants followed up had imputation quality above 0.8 in UK BiLEVE.

The same collapsing methods as in stage 1 were used. The most likely genotype for each individual was used for the analysis, using IMPUTE2 [53] posterior probabilities and including only genotypes with posterior probability > 0.9. Sensitivity analyses were undertaken for the top hits including only independent variants (r^2^ < 0.2) within each locus. In addition, 10,000 permutations (permuting case control status) were run for the C-alpha top hits, only including independent variants, in order to obtain more accurate P-values. The same loci boundaries as in stage 1 were used for the collapsing method including only variants with MAF < 1%, HWE P-value > 10^−6^ and imputation quality ≥ 0.8.

Significance thresholds per region were defined by a Bonferroni corrected threshold for the number of independent tests undertaken in each region. For the single variant analysis the number of independent tests were the number of variants followed up. For the gene based and exon based analyses a gene was considered as an independent test. For the sliding window analysis, two overlapping windows were counted as 1.5 tests.

### Ethics statement

The Gedling study was approved by the Nottingham City Hospital and Nottingham University Ethics committees (MREC/99/4/01) and written informed consent for genetic study was obtained from participants. The Nottingham Smokers study was approved by Nottingham University Medical School Ethical Committee (GM129901/) and written informed consent for genetic study was obtained from participants. For the Leicester COPD cases ethical approval was obtained from the Leicestershire Research Ethics Committee and written informed consent from all subjects was obtained. UK Biobank has approval from the North West Multi-centre Research Ethics Committee (MREC), which covers the UK. It also sought the approval in England and Wales from the Patient Information Advisory Group (PIAG) for gaining access to information that would allow it to invite people to participate. PIAG has since been replaced by the National Information Governance Board for Health & Social Care (NIGB). In Scotland, UK Biobank has approval from the Community Health Index Advisory Group (CHIAG).

## Supporting Information

S1 Fig Venn diagrams of variants called by vipR, SNVer or Syzygy. a) Venn diagrams of SNPs called by any of the three algorithms with and without a 1% minor allele frequency (MAF) filter. The proportion of SNPs included in dbSNP137 [29] for each section is presented in brackets.b) Venn diagrams of indels called by any of the three algorithms with and without 1% MAF filter. The proportion of indels included in 1000 Genomes Project phase 1 data [20] and the proportion included in Mills et al. [30] for each section are presented in brackets in this order.
(DOCX)

S2 Fig Allele frequency comparisons. a) For SNPs, across calling algorithmsb) For SNPs, with 1000 Genomes Project [20]c) For indels, across calling algorithmsd) For indels, with 1000 Genomes Project [20]
(DOCX)

S3 Fig Drop one (top) and single variant association results (bottom) plots. A drop one plot for a locus is obtained by undertaking the C-alpha test removing one variant at a time; the P-value plotted for each variant represented on the x-axis is the P-value obtained after removing that variant. Results obtained using calls from different calling algorithms are represented in different colours, according to the legend in each figure. Not all calling algorithms called the same set of variants. When there is no visible P-value represented for a variant for a given calling algorithm, it is because that variant was not called by that calling algorithm. If a region only includes two variants, no drop one plot is produced, since no C-alpha test can be undertaken with only one variant. Asymptotic P-values are presented here for the C-alpha test.
The single variant plots show the P-values obtained for each variant, and they also show the direction of effect by plotting on the y-axis the -log10 (P-value) x direction of effect, with the direction of effect = 1 if OR > 1 and direction of effect = -1 if OR < 1.
For each region the first row shows drop one plots and the second row shows single variant plots for the same variants; the first column presents results from stage 1 excluding variants not in the joint 1000 Genomes Project [20] and UK10K [21] reference panel (UK10K+1000G), the second column presents results from stage 1 including variants not in this reference panel (marked with * on the x-axis) and the third column presents results from stage 2. Variants that are included both in stage 1 and stage 2 are highlighted in bold. The horizontal dashed line represents the collapsing method significance threshold for each region (note that different thresholds were used for stage 1 and stage 2).
a) chr3:25633833–25636833 (*RARB*)b) chr3:169238286–169241286 (*MECOM*)c) chr4:145293600–145296600 (*HHIP*)d) TNXB (AGER)
(DOCX)

S1 Table Study characteristics. Abbreviations: N = number, sd = standard deviation, y = years, l = litres.
(DOCX)

S2 Table Summary of regions sequenced. “GWAS sentinel” and “GWAS gene” present the lung function GWAS sentinel SNP and the closest gene to the sentinel SNP respectively [9]. Abbreviations: Chr = chromosome.
(DOCX)

S3 Table Significance thresholds in stage 1. Significance thresholds for each region are presented for SNPs and indels for the single variant analysis and for SNPs for the collapsing methods (no indels were included in the collapsing methods analyses). The column “GWAS gene” presents the gene reported in the lung function GWAS [9] for each region. Abbreviations: Chr = chromosome, N = number, UK10K+1000G = joint 1000 Genomes Project and UK10K reference panel.
(DOCX)

S4 Table Single variants associated with COPD risk. a) Stage 1Single variants results for stage 1 are presented for variants that met the criteria for follow-up. “Threshold” and “Threshold support” present the threshold and the threshold for supporting evidence for each region respectively. “GWAS gene” refers to the gene reported in the lung function GWAS [9] for each region. Abbreviations: chr = chromosome, Ref = reference, Alt = alternative, MAF = minor allele frequency and ac = allele countb) Stage 2The column “GWAS gene” presents the gene reported in the lung function GWAS [9] for each region. The OR correspond to the effect on the alternative allele. Abbreviations: chr = chromosome, Ref = reference, Alt = alternative, MAF = minor allele frequency, OR = odds ratio, SE = standard error.
(DOCX)

S5 Table Conditional analysis in *HTR4* region. The column “GWAS gene” presents the gene reported in the lung function GWAS [9] for each region. Abbreviations: chr = chromosome, Ref = reference, Alt = alternative, MAF = minor allele frequency, OR = odds ratio, SE = standard error.
(DOCX)

S6 Table Single variant results for known variants. Results of COPD risk associations for variants previously associated with lung function [7–10] are presented here ordered by chromosome and position and by P-value significance for each variant. P-values < 0.05 are highlighted in bold. “GWAS gene” presents the closest gene to the lung function sentinel SNP reported in [9]. Abbreviations: MAF = minor allele frequency, N alt ac = number of alternative allele counts, N ref ac = number of reference allele counts, freq = frequency, OR = odds ratio.
(DOCX)

S7 Table Collapsing methods stage 1 results meeting the significance threshold. Results are presented for loci (sliding windows or genes) that reach the threshold for follow-up after sensitivity analyses either with (“Independent variants and variants not in UK10K+1000G”) or without (“Independent variants”) including variants not in joint 1000 Genomes Project [20] and UK10K [21] reference panel. Abbreviations: N = number of variants, P = P-value, UK10K+1000G = joint 1000 Genomes Project and UK10K reference panel.
a) Burden test results in stage 1b) C-alpha test results in stage 1
i) Sliding windowii) Gene basediii) Exon based
(DOCX)

S8 Table Collapsing methods stage 2 results. “GWAS gene” is the gene reported in the lung function GWAS [9] for each region. P-values that reach a Bonferroni corrected threshold as defined in the methods section are highlighted in bold.
a) Burden test results in stage 2b) C-alpha test results in stage 2iv) Sliding windowv) Gene basedvi) Exon based
(DOCX)

S1 Supplementary References (DOCX)
