# Supplementary material for: Targeted Sequencing of Lung Function Loci in Chronic Obstructive Pulmonary Disease Cases and Controls
Source: PLoS One. 2017 Jan 23;12(1):e0170222. doi: 10.1371/journal.pone.0170222 (PMC5256917; doi:10.1371/journal.pone.0170222)

## S1 Fig Venn diagrams of variants called by vipR, SNVer or Syzygy

1. Venn diagrams of SNPs called by any of the three algorithms with and without a 1% minor allele frequency (MAF) filter. The proportion of SNPs included in dbSNP137 [[1](#_ENREF_1)] for each section is presented in brackets.


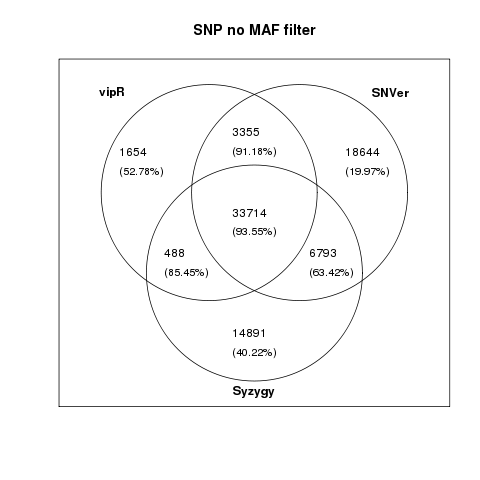

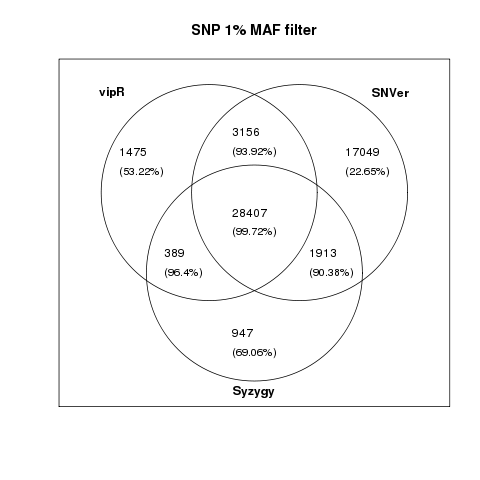


1. Venn diagrams of indels called by any of the three algorithms with and without 1% MAF filter. The proportion of indels included in 1000 Genomes Project phase 1 data [[2](#_ENREF_2)] and the proportion included in Mills et al. [[3](#_ENREF_3)] for each section are presented in brackets in this order.


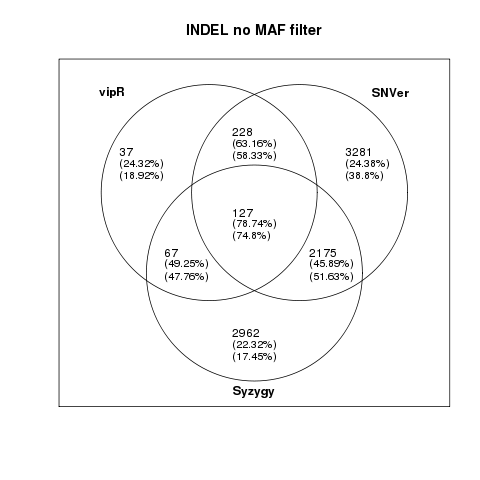

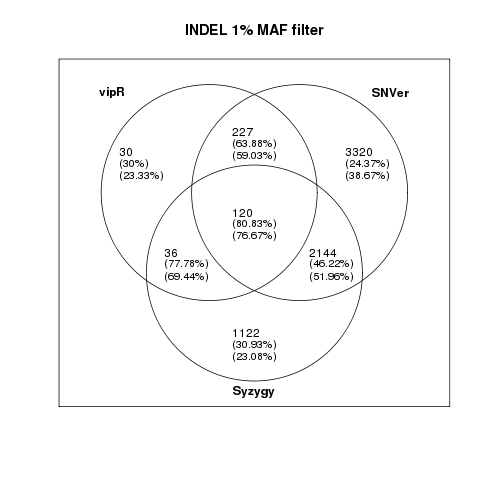

Supplement: S1 Fig — a) Venn diagrams of SNPs called by any of the three algorithms with and without a 1% minor allele frequency (MAF) filter. The proportion of SNPs included in dbSNP137 [29] for each section is presented in brackets.b) Venn diagrams of indels called by any of the three algorithms with and without 1% MAF filter. The proportion of indels included in 1000 Genomes Project phase 1 data [20] and the proportion included in Mills et al. [30] for each section are presented in brackets in this order. (DOCX) [file pone.0170222.s001.docx]
