# Supplementary material for: Targeted Sequencing of Lung Function Loci in Chronic Obstructive Pulmonary Disease Cases and Controls
Source: PLoS One. 2017 Jan 23;12(1):e0170222. doi: 10.1371/journal.pone.0170222 (PMC5256917; doi:10.1371/journal.pone.0170222)

S2 Fig Allele frequency comparisons

1. For SNPs, across calling algorithms


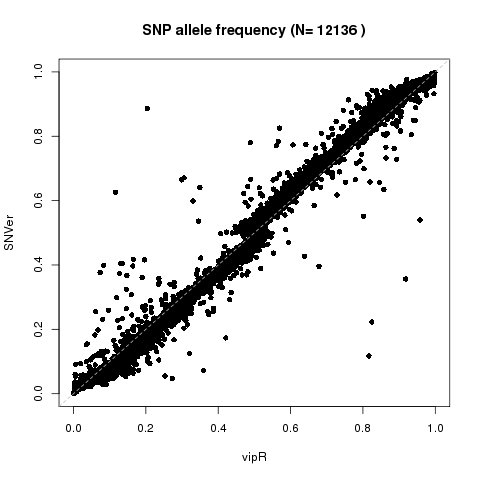

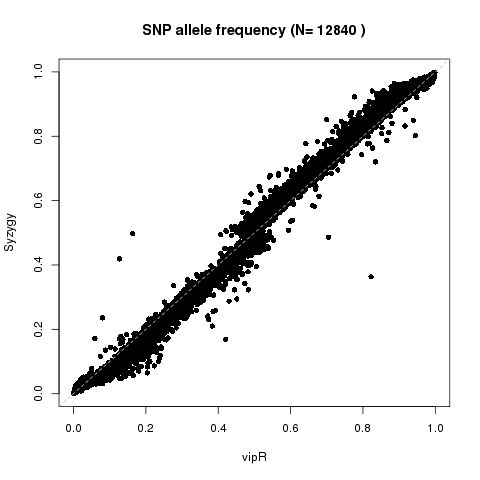

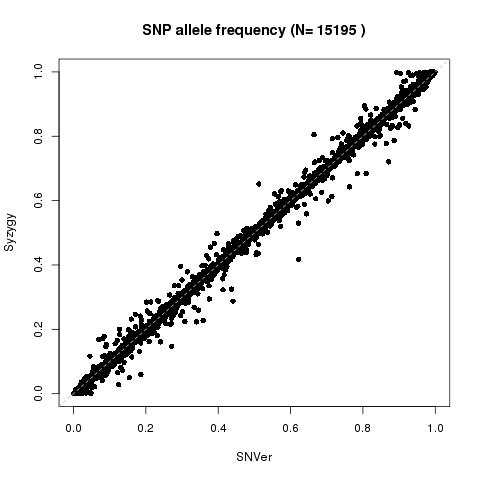


1. For SNPs, with 1000 Genomes Project [[2](#_ENREF_2)]


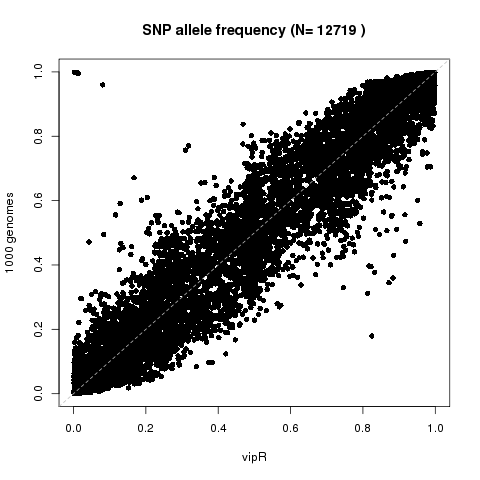

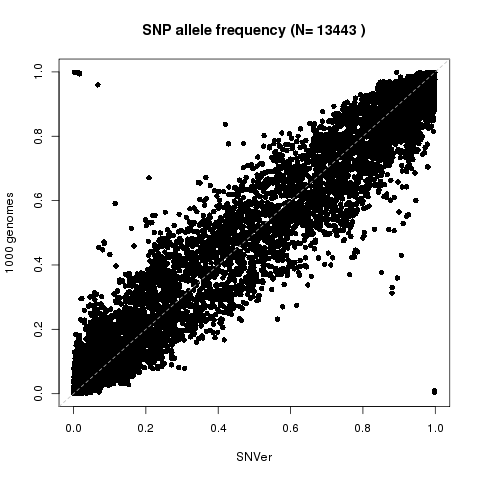

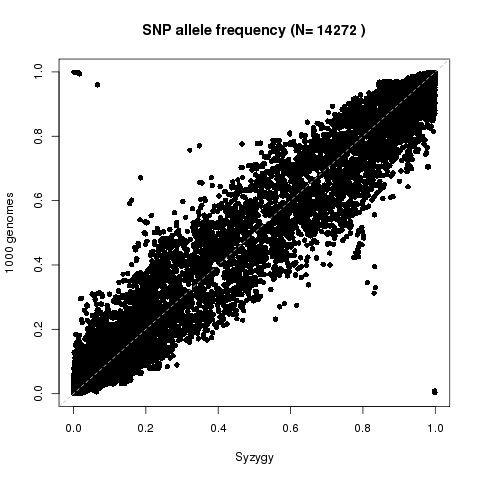


1. For indels, across calling algorithms


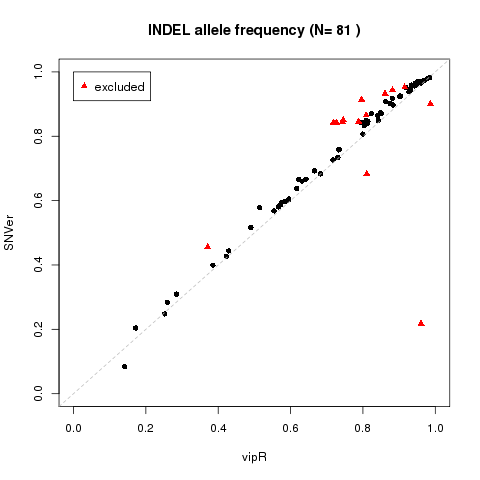

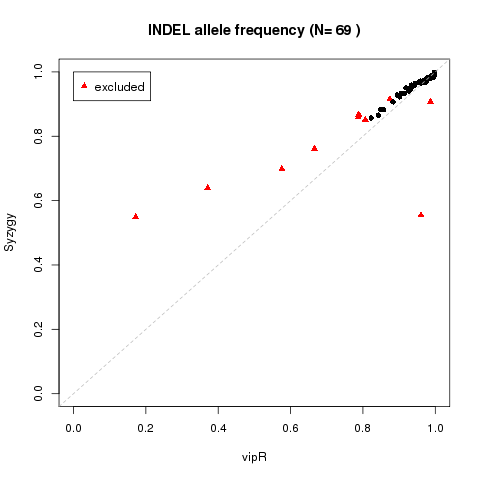

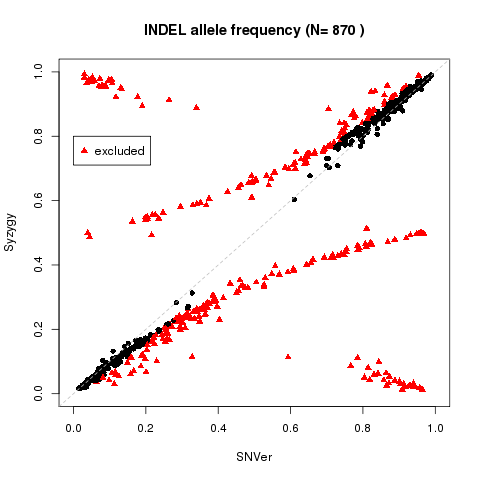


1. For indels, with 1000 Genomes Project [[2](#_ENREF_2)]


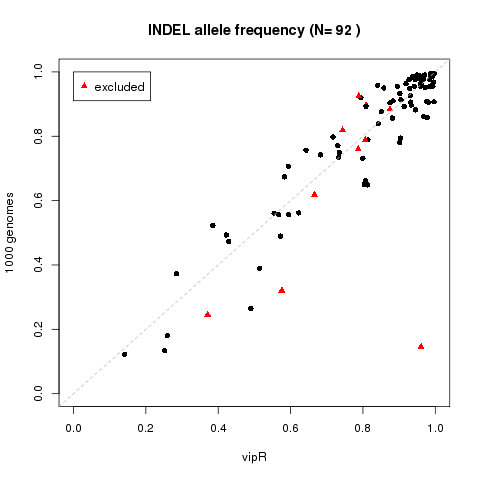

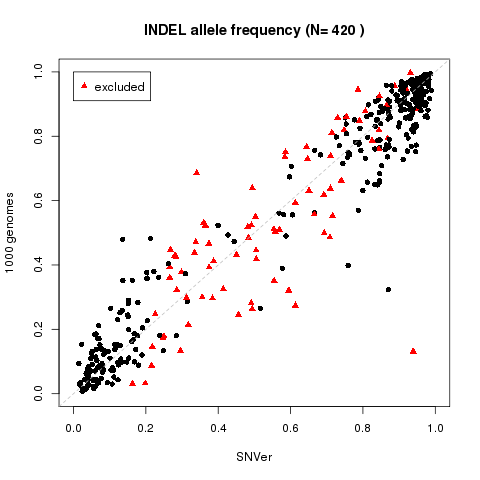

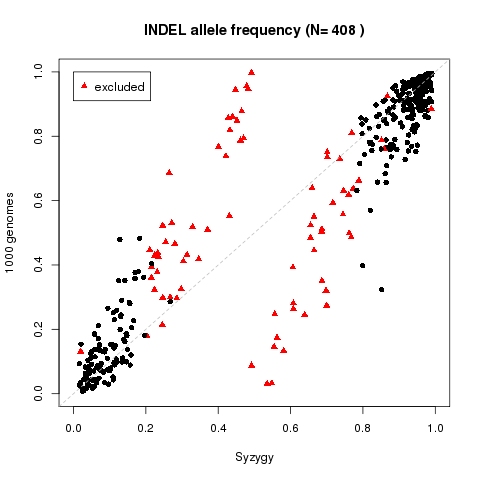

Supplement: S2 Fig — a) For SNPs, across calling algorithmsb) For SNPs, with 1000 Genomes Project [20]c) For indels, across calling algorithmsd) For indels, with 1000 Genomes Project [20] (DOCX) [file pone.0170222.s002.docx]
