# Supplementary material for: Targeted Sequencing of Lung Function Loci in Chronic Obstructive Pulmonary Disease Cases and Controls
Source: PLoS One. 2017 Jan 23;12(1):e0170222. doi: 10.1371/journal.pone.0170222 (PMC5256917; doi:10.1371/journal.pone.0170222)

S3 Fig Drop one (top) and single variant association results (bottom) plots

A drop one plot for a locus is obtained by undertaking the C-alpha test removing one variant at a time; the P-value plotted for each variant represented on the x-axis is the P-value obtained after removing that variant. Results obtained using calls from different calling algorithms are represented in different colours, according to the legend in each figure. Not all calling algorithms called the same set of variants. When there is no visible P-value represented for a variant for a given calling algorithm, it is because that variant was not called by that calling algorithm. If a region only includes two variants, no drop one plot is produced, since no C-alpha test can be undertaken with only one variant. Asymptotic P-values are presented here for the C-alpha test.

The single variant plots show the P-values obtained for each variant, and they also show the direction of effect by plotting on the y-axis the -log10 (P-value) x direction of effect, with the direction of effect=1 if OR > 1 and direction of effect =-1 if OR < 1.

For each region the first row shows drop one plots and the second row shows single variant plots for the same variants; the first column presents results from stage 1 excluding variants not in the joint 1000 Genomes Project [[2](#_ENREF_2)] and UK10K [[4](#_ENREF_4)] reference panel (UK10K+1000G), the second column presents results from stage 1 including variants not in this reference panel (marked with * on the x-axis) and the third column presents results from stage 2. Variants that are included both in stage 1 and stage 2 are highlighted in bold. The horizontal dashed line represents the collapsing method significance threshold for each region (note that different thresholds were used for stage 1 and stage 2).

1. chr3:25633833-25636833 (*RARB*)


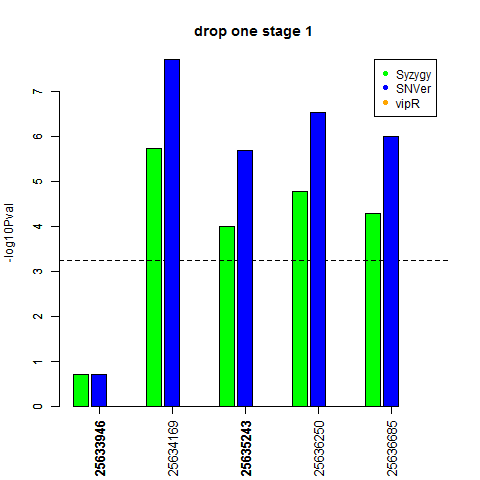

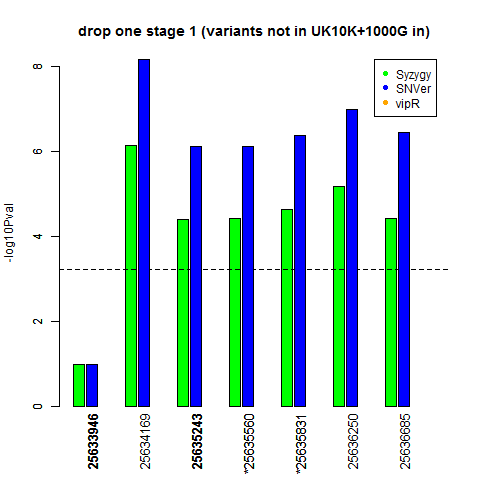

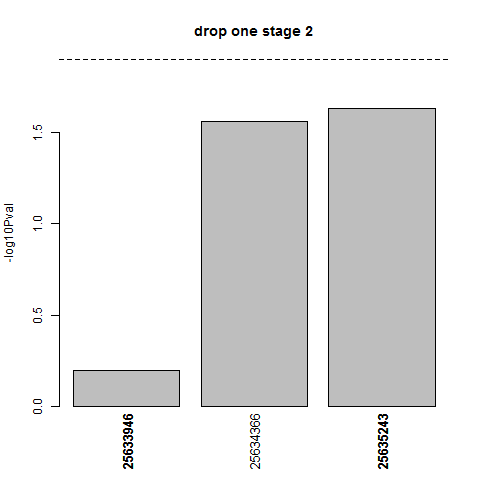


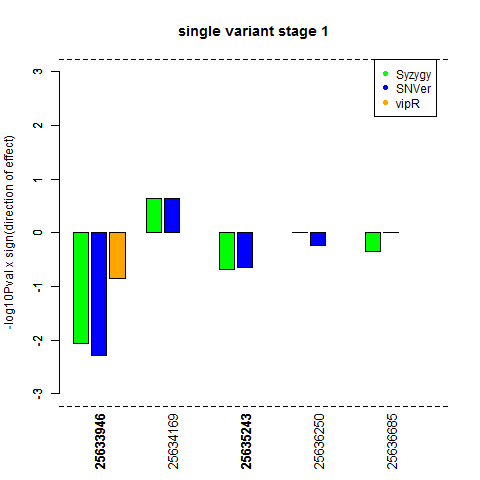

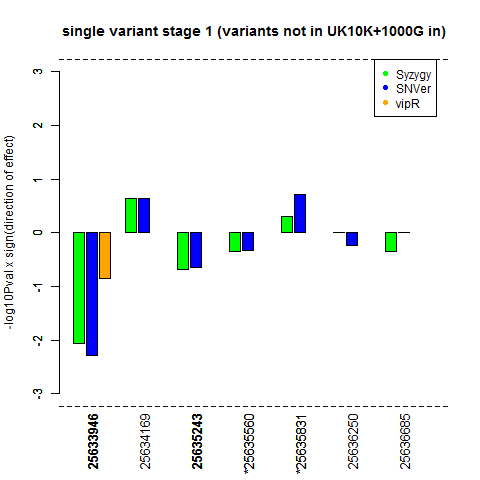

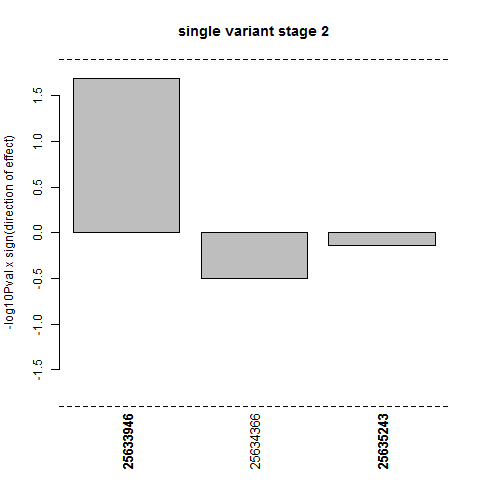


1. chr3:169238286-169241286 (*MECOM*)


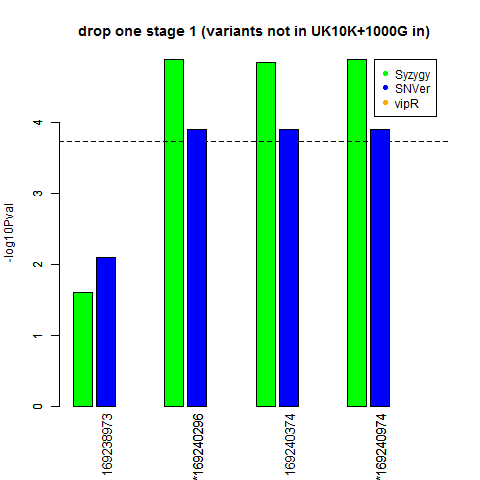

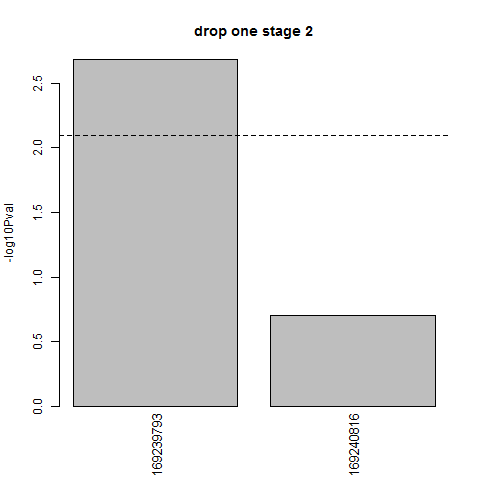


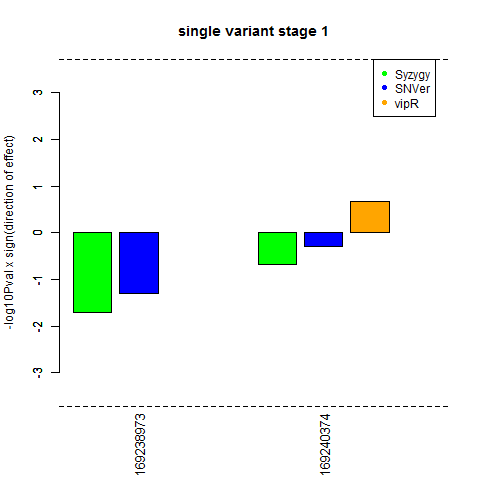

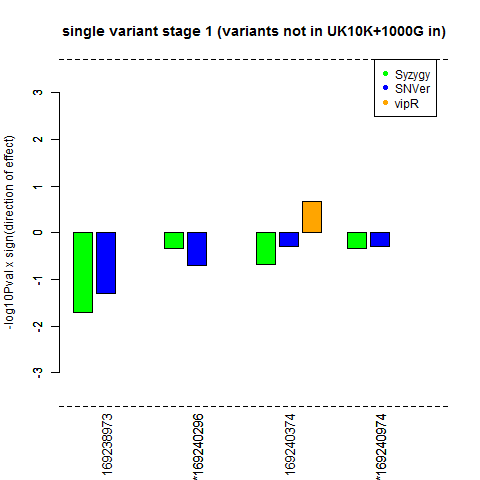

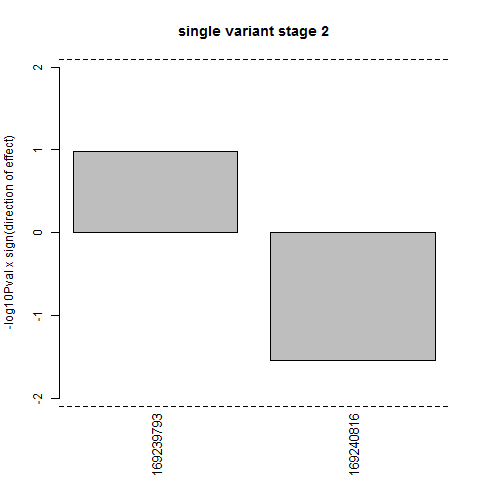


1. chr4:145293600-145296600 (*HHIP*)


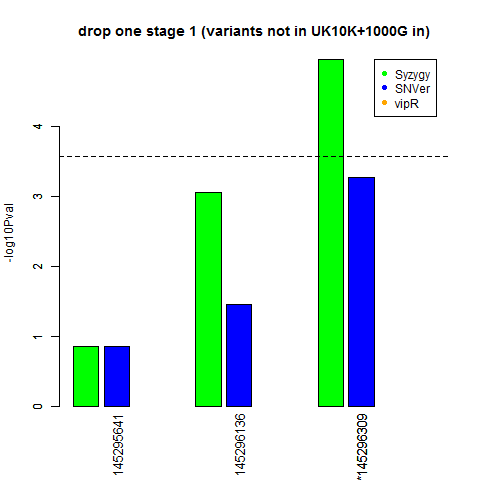

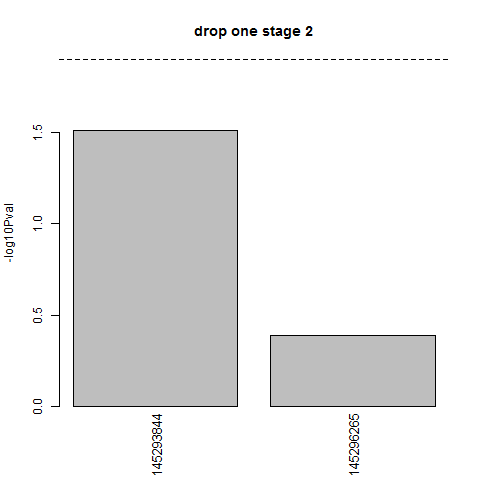


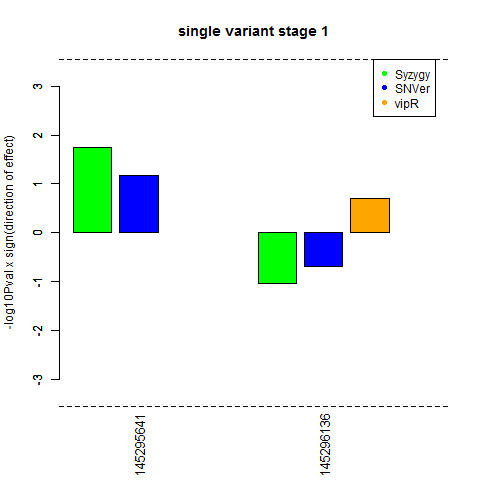

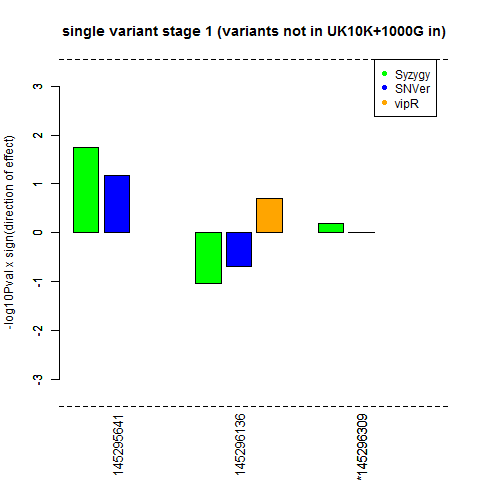

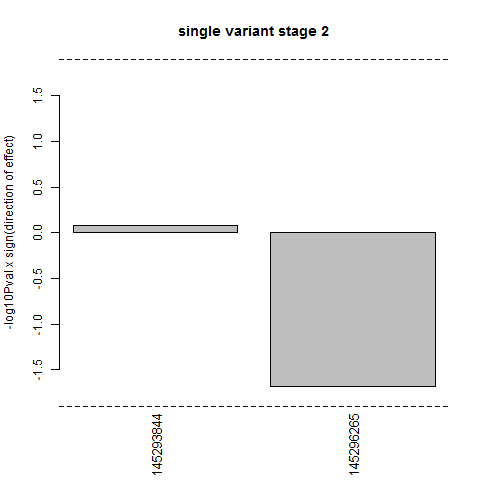


1. *TNXB (AGER)*


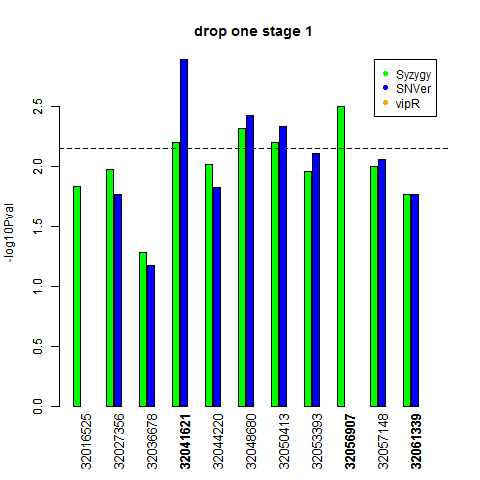

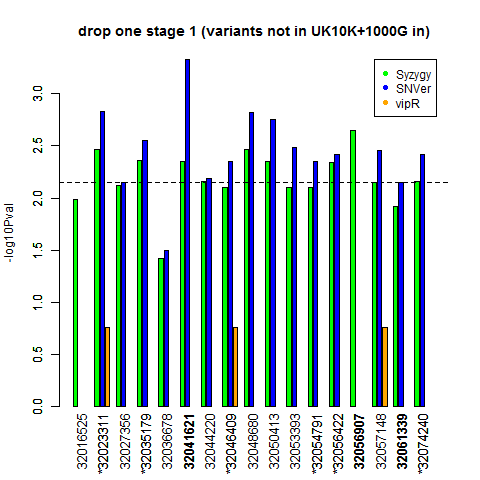

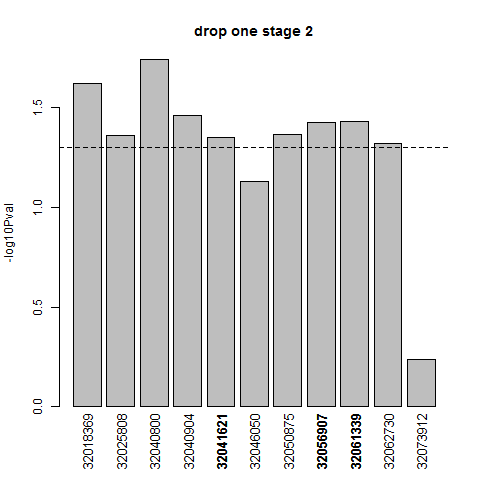


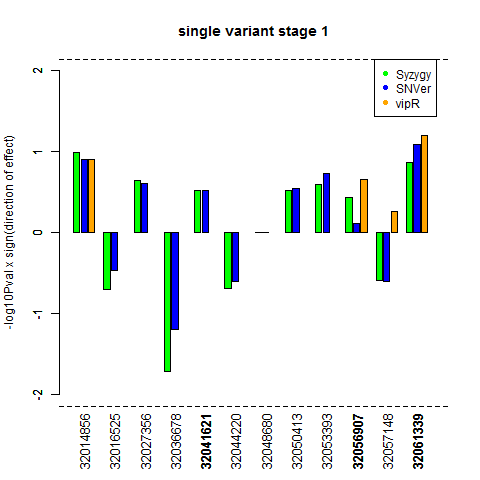

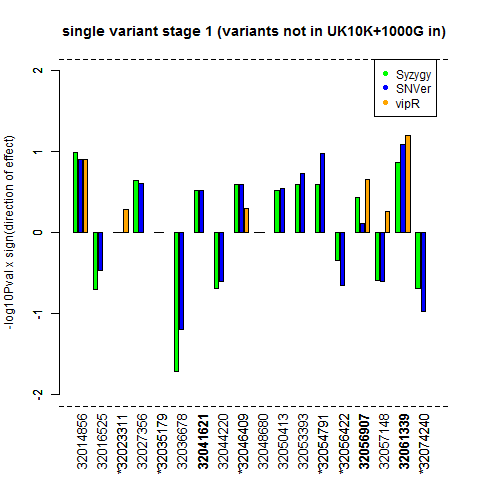

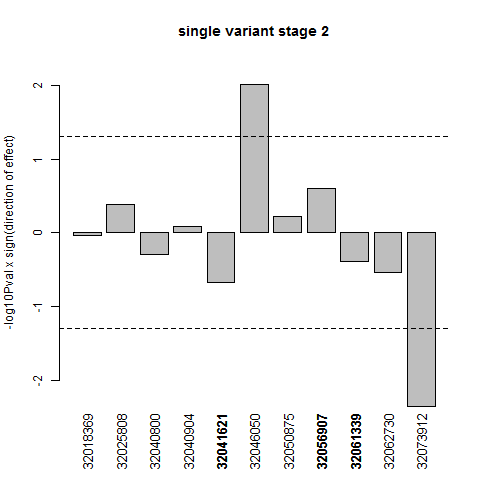

Supplement: S3 Fig — A drop one plot for a locus is obtained by undertaking the C-alpha test removing one variant at a time; the P-value plotted for each variant represented on the x-axis is the P-value obtained after removing that variant. Results obtained using calls from different calling algorithms are represented in different colours, according to the legend in each figure. Not all calling algorithms called the same set of variants. When there is no visible P-value represented for a variant for a given calling algorithm, it is because that variant was not called by that calling algorithm. If a region only includes two variants, no drop one plot is produced, since no C-alpha test can be undertaken with only one variant. Asymptotic P-values are presented here for the C-alpha test. The single variant plots show the P-values obtained for each variant, and they also show the direction of effect by plotting on the y-axis the -log10 (P-value) x direction of effect, with the direction of effect = 1 if OR > 1 and direction of effect = -1 if OR < 1. For each region the first row shows drop one plots and the second row shows single variant plots for the same variants; the first column presents results from stage 1 excluding variants not in the joint 1000 Genomes Project [20] and UK10K [21] reference panel (UK10K+1000G), the second column presents results from stage 1 including variants not in this reference panel (marked with * on the x-axis) and the third column presents results from stage 2. Variants that are included both in stage 1 and stage 2 are highlighted in bold. The horizontal dashed line represents the collapsing method significance threshold for each region (note that different thresholds were used for stage 1 and stage 2). a) chr3:25633833–25636833 (RARB)b) chr3:169238286–169241286 (MECOM)c) chr4:145293600–145296600 (HHIP)d) TNXB (AGER) (DOCX) [file pone.0170222.s003.docx]
