# Supplementary material for: Targeted Sequencing of Lung Function Loci in Chronic Obstructive Pulmonary Disease Cases and Controls
Source: PLoS One. 2017 Jan 23;12(1):e0170222. doi: 10.1371/journal.pone.0170222 (PMC5256917; doi:10.1371/journal.pone.0170222)
Supplement: S1 Table — Abbreviations: N = number, sd = standard deviation, y = years, l = litres. (DOCX) [file pone.0170222.s004.docx]

## S1 Table Study characteristics

Abbreviations: N=number, sd=standard deviation, y=years, l=litres.

| **Status** | **N total** | **N male** | **N female** | **Age range (y)** | **Mean age, y (sd)** | **Mean FEV_1_, l (sd)** | **Mean % predicted FEV_1_ (sd)** | **Mean FVC, l (sd)** | **Mean FEV_1_/ FVC (sd)** | **Mean Pack-years (sd)** |
| --- | --- | --- | --- | --- | --- | --- | --- | --- | --- | --- |
| Stage 1 | | | | | | | | | | |
| Case | 300 | 192 | 108 | 40-86 | 65.35 (9.61) | 1.15 (0.48) | 39.8 (12.96) | 2.42 (0.78) | 0.47 (0.12) | 41.94 (19.18) |
| Control | 300 | 162 | 138 | 40-79 | 56.89 (9.97) | 3.04 (0.68) | 99.27 (8.02) | 3.90 (0.86) | 0.78 (0.04) | 24.69 (16.37) |
| Stage 2 | | | | | | | | | | |
| Case | 4,249 | 2,371 | 1,878 | 40-70 | 59.49 (6.74) | 1.81 (0.52) | 61.82 (11.48) | 2.97 (0.78) | 0.61 (0.07) | 40.72 (17.21) |
| Control | 11,916 | 5,753 | 6,163 | 40-70 | 55.94 (8.09) | 2.98 (0.73) | 100.51 (14.55) | 3.86 (0.93) | 0.77 (0.04) | 30.76 (13.59) |
