# Supplementary material for: Targeted Sequencing of Lung Function Loci in Chronic Obstructive Pulmonary Disease Cases and Controls
Source: PLoS One. 2017 Jan 23;12(1):e0170222. doi: 10.1371/journal.pone.0170222 (PMC5256917; doi:10.1371/journal.pone.0170222)
Supplement: S2 Table — “GWAS sentinel” and “GWAS gene” present the lung function GWAS sentinel SNP and the closest gene to the sentinel SNP respectively [9]. Abbreviations: Chr = chromosome. (DOCX) [file pone.0170222.s005.docx]

S2 Table Summary of the regions sequenced

“GWAS sentinel” and “GWAS gene” present the lung function GWAS sentinel SNP and the closest gene to the sentinel SNP respectively [[5](#_ENREF_5)]. Abbreviations: Chr= chromosome.

| **Chr** | **GWAS sentinel** | **GWAS gene** | **Start** | **End** | **Length (bp)** | **Number of genes** |
| --- | --- | --- | --- | --- | --- | --- |
| 1 | rs2284746 | *MFAP2* | 17238444 | 17455948 | 217504 | 5 |
| 1 | rs993925 | *TGFB2* | 218508675 | 218885482 | 376807 | 2 |
| 2 | rs2571445 | *TNS1* | 218627794 | 218818796 | 191002 | 1 |
| 2 | rs12477314 | *HDAC4* | 239839616 | 240332643 | 493027 | 2 |
| 3 | rs1529672 | *RARB* | 25459833 | 25649422 | 189589 | 2 |
| 3 | rs1344555 | *MECOM* | 168791286 | 169391563 | 600277 | 1 |
| 4 | rs2045517 | *FAM13A* | 89637105 | 90077431 | 440326 | 2 |
| 4 | rs10516526 | *GSTCD* | 106280233 | 106902828 | 622595 | 5 |
| 4 | rs11100860 | *HHIP* | 145227600 | 145669881 | 442281 | 1 |
| 5 | rs153916 | *SPATA9* | 94984019 | 95038027 | 54008 | 2 |
| 5 | rs1985524 | *HTR4* | 147682118 | 148026624 | 344506 | 4 |
| 5 | rs11134779 | *ADAM19* | 156597906 | 157139503 | 541597 | 7 |
| 6 | rs6903823 | *ZKSCAN3* | 27982152 | 28415572 | 433420 | 14 |
| 6 | rs2857595 | *NCR3* | 30584612 | 31959223 | 1374611 | 75 |
| 6 | rs2070600 | *AGER* | 31996092 | 32205942 | 209850 | 14 |
| 6 | rs2798641 | *ARMC2* | 109159618 | 109305352 | 145734 | 1 |
| 6 | rs262129 | *LOC153910* | 142613055 | 142968973 | 355918 | 2 |
| 9 | rs16909859 | *PTCH1* | 98153197 | 98313032 | 159835 | 1 |
| 10 | rs7068966 | *CDC123* | 12170174 | 12335588 | 165414 | 4 |
| 10 | rs11001819 | *C10orf11* | 77532518 | 78643886 | 1111368 | 1 |
| 12 | rs11172113 | *LRP1* | 57472676 | 57617125 | 144449 | 4 |
| 12 | rs1036429 | *CCDC38* | 96041582 | 96400071 | 358489 | 6 |
| 15 | rs8033889 | *THSD4* | 71423787 | 72085722 | 661935 | 1 |
| 16 | rs12447804 | *MMP15* | 57906243 | 58143392 | 237149 | 5 |
| 16 | rs2865531 | *CFDP1* | 75252927 | 75538926 | 285999 | 5 |
| 21 | rs9978142 | *KCNE2* | 35595821 | 35753440 | 157619 | 2 |
