# Supplementary material for: Targeted Sequencing of Lung Function Loci in Chronic Obstructive Pulmonary Disease Cases and Controls
Source: PLoS One. 2017 Jan 23;12(1):e0170222. doi: 10.1371/journal.pone.0170222 (PMC5256917; doi:10.1371/journal.pone.0170222)
Supplement: S3 Table — Significance thresholds for each region are presented for SNPs and indels for the single variant analysis and for SNPs for the collapsing methods (no indels were included in the collapsing methods analyses). The column “GWAS gene” presents the gene reported in the lung function GWAS [9] for each region. Abbreviations: Chr = chromosome, N = number, UK10K+1000G = joint 1000 Genomes Project and UK10K reference panel. (DOCX) [file pone.0170222.s006.docx]

S3 Table Significance thresholds in stage 1

Significance thresholds for each region are presented for SNPs and indels for the single variant analysis and for SNPs for the collapsing methods (no indels were included in the collapsing methods analyses).The column “GWAS gene” presents the gene reported in the lung function GWAS [[5](#_ENREF_5)] for each region. Abbreviations: Chr=chromosome, N=number, UK10K+1000G=joint 1000 Genomes Project and UK10K reference panel.

| **Chr: start-end** | **GWAS gene** | **Variant type** | **Collapsing methods thresholds** | | | **N variants** | **N variants in UK10K+**  **1000G** | **N tests in**  **UK10K+1000G** | **N tests**  **final** | **Single variant thresholds** |
| --- | --- | --- | --- | --- | --- | --- | --- | --- | --- | --- |
|  |  |  | **Gene based** | **Exon based** | **Slide**  **window** |  |  |  |  |  |
| chr1:17238444-17455948 | *MFAP2* | SNP | 1.25x${10}^{-2}$ | 1.67x${10}^{-2}$ | 1.72x${10}^{-3}$ | 338 | 291 | 99 | 146 | 3.43x${10}^{-4}$ |
|  |  | indel | - | - | - | 9 | 5 | 5 | 9 | 5.56x${10}^{-3}$ |
| chr1:218508675-218885482 | *TGFB2* | SNP | 5x${10}^{-2}$ | 5x${10}^{-2}$ | 4.24x${10}^{-4}$ | 691 | 612 | 248 | 327 | 1.53x${10}^{-4}$ |
|  |  | indel | - | - | - | 36 | 23 | 18 | 31 | 1.61x${10}^{-3}$ |
| chr2:218627794-218818796 | *TNS1* | SNP | 5x${10}^{-2}$ | 5x${10}^{-2}$ | 1.09x${10}^{-3}$ | 337 | 309 | 146 | 174 | 2.87x${10}^{-4}$ |
|  |  | indel | - | - | - | 5 | 5 | 5 | 5 | 1x${10}^{-2}$ |
| chr2:239839616-240332643 | *HDAC4* | SNP | 5x${10}^{-2}$ | 5x${10}^{-2}$ | 3.68x${10}^{-4}$ | 1243 | 1159 | 396 | 480 | 1.04x${10}^{-4}$ |
|  |  | indel | - | - | - | 34 | 26 | 21 | 29 | 1.72x${10}^{-3}$ |
| chr3:25459833-25649422 | *RARB* | SNP | 2.5x${10}^{-2}$ | 2.5x${10}^{-2}$ | 5.81x${10}^{-4}$ | 471 | 414 | 200 | 257 | 1.95x${10}^{-4}$ |
|  |  | indel | - | - | - | 24 | 18 | 14 | 20 | 2.5x${10}^{-3}$ |
| chr3:168791286-169391563 | *MECOM* | SNP | 5x${10}^{-2}$ | 5x${10}^{-2}$ | 1.87x${10}^{-4}$ | 1326 | 1152 | 460 | 634 | 7.89x${10}^{-5}$ |
|  |  | indel | - | - | - | 54 | 37 | 29 | 46 | 1.09x${10}^{-3}$ |
| chr4:89637105-90077431 | *FAM13A* | SNP | 5x${10}^{-2}$ | 5x${10}^{-2}$ | 5x${10}^{-4}$ | 666 | 591 | 200 | 275 | 1.82x${10}^{-4}$ |
|  |  | indel | - | - | - | 31 | 24 | 18 | 25 | 2x${10}^{-3}$ |
| chr4:106280233-106902828 | *GSTCD* | SNP | 1x${10}^{-2}$ | 1.25x${10}^{-2}$ | 2.69x${10}^{-4}$ | 1031 | 922 | 328 | 437 | 1.14x${10}^{-4}$ |
|  |  | indel | - | - | - | 58 | 37 | 20 | 41 | 1.22x${10}^{-3}$ |
| chr4:145227600-145669881 | *HHIP* | SNP | 5x${10}^{-2}$ | 5x${10}^{-2}$ | 2.76x${10}^{-4}$ | 802 | 686 | 248 | 364 | 1.37x${10}^{-4}$ |
|  |  | indel | - | - | - | 25 | 15 | 12 | 22 | 2.27x${10}^{-3}$ |
| chr5:94984019-95038027 | *SPATA9* | SNP | 2.5x${10}^{-2}$ | - | 2.5x${10}^{-3}$ | 77 | 69 | 42 | 50 | 1x${10}^{-3}$ |
|  |  | indel | - | - | - | 2 | 2 | 2 | 2 | 2.5x${10}^{-2}$ |
| chr5:147682118-148026624 | *HTR4* | SNP | 1.25x${10}^{-2}$ | 1.67x${10}^{-2}$ | 4.9x${10}^{-4}$ | 468 | 414 | 197 | 251 | 1.99x${10}^{-4}$ |
|  |  | indel | - | - | - | 17 | 13 | 7 | 11 | 4.55x${10}^{-3}$ |
| chr5:156597906-157139503 | *ADAM19* | SNP | 8.33x${10}^{-3}$ | 1.25x${10}^{-2}$ | 5.88x${10}^{-4}$ | 670 | 615 | 236 | 291 | 1.72x${10}^{-4}$ |
|  |  | indel | - | - | - | 25 | 20 | 17 | 22 | 2.27x${10}^{-3}$ |
| chr6:27982152-28415572 | *ZKSCAN3* | SNP | 3.85x${10}^{-3}$ | 1x${10}^{-2}$ | 4.39x${10}^{-4}$ | 520 | 459 | 162 | 223 | 2.24x${10}^{-4}$ |
|  |  | indel | - | - | - | 26 | 23 | 10 | 13 | 3.85x${10}^{-3}$ |
| chr6:30584612-31959223 | *NCR3* | SNP | 1.22x${10}^{-3}$ | 3.13x${10}^{-3}$ | 2.79x${10}^{-4}$ | 3507 | 3307 | 647 | 847 | 5.9x${10}^{-5}$ |
|  |  | indel | - | - | - | 115 | 98 | 54 | 71 | 7.04x${10}^{-4}$ |
| chr6:31996092-32205942 | *AGER* | SNP | 7.14x${10}^{-3}$ | 2.5x${10}^{-2}$ | 2.17x${10}^{-3}$ | 283 | 270 | 87 | 100 | 5x${10}^{-4}$ |
|  |  | indel | - | - | - | 14 | 11 | 10 | 13 | 3.85x${10}^{-3}$ |
| chr6:109159618-109305352 | *ARMC2* | SNP | 5x${10}^{-2}$ | 5x${10}^{-2}$ | 1.11x${10}^{-3}$ | 213 | 189 | 97 | 121 | 4.13x${10}^{-4}$ |
|  |  | indel | - | - | - | 9 | 7 | 6 | 8 | 6.25x${10}^{-3}$ |
| chr6:142613055-142968973 | *GPR126* | SNP | 2.5x${10}^{-2}$ | 5x${10}^{-2}$ | 4.27x${10}^{-4}$ | 443 | 388 | 196 | 251 | 1.99x${10}^{-4}$ |
|  |  | indel | - | - | - | 20 | 9 | 6 | 17 | 2.94x${10}^{-3}$ |
| chr9:98153197-98313032 | *PTCH1* | SNP | 5x${10}^{-2}$ | - | 9.8x${10}^{-4}$ | 226 | 200 | 96 | 122 | 4.1x${10}^{-4}$ |
|  |  | indel | - | - | - | 13 | 8 | 6 | 11 | 4.55x${10}^{-3}$ |
| chr10:12170174-12335588 | *CDC123* | SNP | 1.67x${10}^{-2}$ | 5x${10}^{-2}$ | 1.09x${10}^{-3}$ | 226 | 192 | 91 | 125 | 4x${10}^{-4}$ |
|  |  | indel | - | - | - | 11 | 4 | 4 | 11 | 4.55x${10}^{-3}$ |
| chr10:77532518-78643886 | *C10orf11* | SNP | 5x${10}^{-2}$ | - | 1.71x${10}^{-4}$ | 1513 | 1336 | 535 | 712 | 7.02x${10}^{-5}$ |
|  |  | indel | - | - | - | 31 | 17 | 15 | 29 | 1.72x${10}^{-3}$ |
| chr12:57472676-57617125 | *LRP1* | SNP | 1.25x${10}^{-2}$ | 2.5x${10}^{-2}$ | 1.56x${10}^{-3}$ | 169 | 155 | 87 | 101 | 4.95x${10}^{-4}$ |
|  |  | indel | - | - | - | 2 | 2 | 2 | 2 | 2.5x${10}^{-2}$ |
| chr12:96041582-96400071 | *CCDC38* | SNP | 8.33x${10}^{-3}$ | 1.67x${10}^{-2}$ | 5.05x${10}^{-4}$ | 651 | 586 | 216 | 281 | 1.78x${10}^{-4}$ |
|  |  | indel | - | - | - | 26 | 21 | 18 | 23 | 2.17x${10}^{-3}$ |
| chr15:71423787-72085722 | *THSD4* | SNP | 5x${10}^{-2}$ | 5x${10}^{-2}$ | 2.66x${10}^{-4}$ | 1266 | 1151 | 446 | 561 | 8.91x${10}^{-5}$ |
|  |  | indel | - | - | - | 32 | 19 | 18 | 31 | 1.61x${10}^{-3}$ |
| chr16:57906243-58143392 | *MMP15* | SNP | 1x${10}^{-2}$ | 1.67x${10}^{-2}$ | 1.61x${10}^{-3}$ | 310 | 288 | 130 | 152 | 3.29x${10}^{-4}$ |
|  |  | indel | - | - | - | 9 | 4 | 4 | 9 | 5.56x${10}^{-3}$ |
| chr16:75252927-75538926 | *CFDP1* | SNP | 1x${10}^{-2}$ | 2.5x${10}^{-2}$ | 5.88x${10}^{-4}$ | 517 | 481 | 177 | 213 | 2.35x${10}^{-4}$ |
|  |  | indel | - | - | - | 7 | 2 | 2 | 7 | 7.14x${10}^{-3}$ |
| chr21:35595821-35753440 | *KCNE2* | SNP | 2.5x${10}^{-2}$ | 5x${10}^{-2}$ | 1.25x${10}^{-3}$ | 213 | 190 | 108 | 131 | 3.82x${10}^{-4}$ |
|  |  | indel | - | - | - | 8 | 6 | 6 | 8 | 6.25x${10}^{-3}$ |
