# Supplementary material for: Targeted Sequencing of Lung Function Loci in Chronic Obstructive Pulmonary Disease Cases and Controls
Source: PLoS One. 2017 Jan 23;12(1):e0170222. doi: 10.1371/journal.pone.0170222 (PMC5256917; doi:10.1371/journal.pone.0170222)
Supplement: S4 Table — a) Stage 1Single variants results for stage 1 are presented for variants that met the criteria for follow-up. “Threshold” and “Threshold support” present the threshold and the threshold for supporting evidence for each region respectively. “GWAS gene” refers to the gene reported in the lung function GWAS [9] for each region. Abbreviations: chr = chromosome, Ref = reference, Alt = alternative, MAF = minor allele frequency and ac = allele countb) Stage 2The column “GWAS gene” presents the gene reported in the lung function GWAS [9] for each region. The OR correspond to the effect on the alternative allele. Abbreviations: chr = chromosome, Ref = reference, Alt = alternative, MAF = minor allele frequency, OR = odds ratio, SE = standard error. (DOCX) [file pone.0170222.s007.docx]

S4 Table Single variants associated with COPD risk

1. Stage 1

Single variants results for stage 1 are presented for variants that met the criteria for follow-up. “Threshold” and “Threshold support” present the threshold and the threshold for supporting evidence for each region respectively. “GWAS gene” refers to the gene reported in the lung function GWAS [[5](#_ENREF_5)] for each region. Abbreviations: chr=chromosome, Ref=reference, Alt= alternative, MAF=minor allele frequency and ac=allele count

| **rs number (chr: position), function** | **GWAS gene** | **Ref allele** | **Alt allele** | **Threshold** | **Threshold support** | **Calling algorithm** | **MAF** | **Alt ac cases** | **Alt ac controls** | **P-value** |
| --- | --- | --- | --- | --- | --- | --- | --- | --- | --- | --- |
| rs11678706 (chr2:239908773), intergenic | *HDAC4* | A | C | 1.04x${10}^{-4}$ | 2.08x${10}^{-4}$ | vipR | 0.219 | 0.221 | 0.203 | 6.92x${10}^{-1}$ |
|  |  |  |  |  |  | SNVer | 0.158 | 0.200 | 0.101 | 8.26x${10}^{-6}$ |
|  |  |  |  |  |  | Syzygy | 0.159 | 0.208 | 0.105 | 1.87x${10}^{-4}$ |
| rs16854211 (chr3:169338409), intronic (*MECOM*) | *MECOM* | G | T | 7.89x${10}^{-5}$ | 1.58x${10}^{-4}$ | vipR | 0.169 | 0.194 | 0.124 | 4.23x${10}^{-3}$ |
|  |  |  |  |  |  | SNVer | 0.159 | 0.195 | 0.107 | 5.29x${10}^{-5}$ |
|  |  |  |  |  |  | Syzygy | 0.155 | 0.198 | 0.108 | 7.92x${10}^{-5}$ |
| rs1895031 (chr3:169354498), intronic (*MECOM*) | *MECOM* | G | C | 7.89x${10}^{-5}$ | 1.58x${10}^{-4}$ | vipR | 0.291 | 0.250 | 0.346 | 9.00x${10}^{-4}$ |
|  |  |  |  |  |  | SNVer | 0.289 | 0.244 | 0.355 | 5.00x${10}^{-5}$ |
|  |  |  |  |  |  | Syzygy | 0.298 | 0.246 | 0.357 | 1.58x${10}^{-4}$ |
| rs193259319 (chr5:147823559), downstream (*FBXO38*) | *HTR4* | T | C | 1.99x${10}^{-4}$ | 3.98x${10}^{-4}$ | vipR | 0.043 | 0.068 | 0.006 | 2.56x${10}^{-5}$ |
|  |  |  |  |  |  | SNVer | 0.039 | 0.060 | 0.012 | 1.26x${10}^{-4}$ |
|  |  |  |  |  |  | Syzygy | 0.033 | 0.048 | 0.011 | 2.9x${10}^{-4}$ |
| rs999741 (chr5:147727048), transcript (*RP11-373N22.3*) | *HTR4* | C | G | 1.99x${10}^{-4}$ | 3.98x${10}^{-4}$ | vipR | 0.254 | 0.219 | 0.288 | 2.15x${10}^{-2}$ |
|  |  |  |  |  |  | SNVer | 0.231 | 0.188 | 0.291 | 6.24x${10}^{-5}$ |
|  |  |  |  |  |  | Syzygy | 0.235 | 0.192 | 0.294 | 3.53x${10}^{-4}$ |
| rs138649528 (chr6:30776469), downstream (*NCR-00243*) | *NCR3* | AT | A | 7.04x${10}^{-4}$ | 1.41x${10}^{-3}$ | vipR | - | - | - | - |
|  |  |  |  |  |  | SNVer | 0.032 | 0.014 | 0.058 | 1.02x${10}^{-4}$ |
|  |  |  |  |  |  | Syzygy | 0.033 | 0.013 | 0.055 | 1.11x${10}^{-3}$ |
| rs35278224;rs67982043 (chr6:32164665), intronic (*NOTCH4*) | *AGER* | CT | C | 3.85x${10}^{-3}$ | 7.69x${10}^{-3}$ | vipR | - | - | - | - |
|  |  |  |  |  |  | SNVer | 0.045 | 0.022 | 0.078 | 1.2x${10}^{-5}$ |
|  |  |  |  |  |  | Syzygy | 0.046 | 0.023 | 0.073 | 1.24x${10}^{-3}$ |
| rs146088795 (chr6:142640832), intronic (*GPR126*) | *GPR126* | A | G | 1.99x${10}^{-4}$ | 3.98x${10}^{-4}$ | vipR | 0.011 | 0.000 | 0.048 | 1.98x${10}^{-4}$ |
|  |  |  |  |  |  | SNVer | 0.018 | 0.007 | 0.032 | 4.08x${10}^{-3}$ |
|  |  |  |  |  |  | Syzygy | 0.018 | 0.006 | 0.032 | 3.73x${10}^{-4}$ |
| rs7174934 (chr15:71571345), intronic (*THSD4*) | *THSD4* | G | A | 8.91x${10}^{-5}$ | 1.78x${10}^{-4}$ | vipR | 0.432 | 0.382 | 0.495 | 3.54x${10}^{-4}$ |
|  |  |  |  |  |  | SNVer | 0.423 | 0.368 | 0.501 | 1.03x${10}^{-5}$ |
|  |  |  |  |  |  | Syzygy | 0.434 | 0.381 | 0.510 | 9.18x${10}^{-5}$ |
| rs75958385 (chr16:75403497), intronic (*CFDP1*) | *CFDP1* | G | A | 2.35x${10}^{-4}$ | 4.7x${10}^{-4}$ | vipR | 0.013 | 0.000 | 0.047 | 4.16x${10}^{-4}$ |
|  |  |  |  |  |  | SNVer | 0.015 | 0.004 | 0.029 | 2.75x${10}^{-3}$ |
|  |  |  |  |  |  | Syzygy | 0.014 | 0.003 | 0.026 | 2.31x${10}^{-4}$ |
| rs199588075 (chr21:35679578), transcript (*AP000318.2*) | *KCNE2* | CT | C | 6.25x${10}^{-3}$ | 1.25x${10}^{-2}$ | vipR | 0.048 | 0.073 | 0 | 1.55x${10}^{-5}$ |
|  |  |  |  |  |  | SNVer | 0.034 | 0.048 | 0.014 | 1.78x${10}^{-3}$ |
|  |  |  |  |  |  | Syzygy | - | - | - | - |

1. Stage 2

The column “GWAS gene” presents the gene reported in the lung function GWAS [[5](#_ENREF_5)] for each region. The OR correspond to the effect on the alternative allele. Abbreviations: chr=chromosome, Ref=reference, Alt=alternative, MAF=minor allele frequency, OR=odds ratio, SE=standard error.

| **rs number (chr: position), function** | **GWAS gene** | **Ref allele** | **Alt allele** | **MAF** | **Imputation information** | **OR** | **SE** | **P-value** | **Consistent direction of effect?** |
| --- | --- | --- | --- | --- | --- | --- | --- | --- | --- |
| rs11678706 (chr2:239908773), intergenic | *HDAC4* | A | C | 0.175 | 0.986 | 0.953 | 0.034 | 1.52x${10}^{-1}$ | NO |
| rs16854211 (chr3:169338409), intronic (*MECOM*) | *MECOM* | G | T | 0.156 | 0.977 | 1.058 | 0.035 | 1.07x${10}^{-1}$ | YES |
| rs1895031 (chr3:169354498), intronic (*MECOM*) | *MECOM* | G | C | 0.295 | 1 | 0.987 | 0.028 | 6.5x${10}^{-1}$ | YES |
| rs999741 (chr5:147727048), transcript (*RP11-373N22.3*) | *HTR4* | C | G | 0.256 | 0.999 | 0.915 | 0.029 | 2x${10}^{-3}$ | YES |
| rs193259319 (chr5:147823559), downstream (*FBXO38*) | *HTR4* | T | C | 0.021 | 1 | 1.053 | 0.085 | 5.46x${10}^{-1}$ | YES |
| rs138649528 (chr6:30776469), downstream (*NCR-00243*) | *NCR3* | AT | A | 0.028 | 0.999 | 0.87 | 0.073 | 5.7x${10}^{-2}$ | NO |
| rs35278224;rs67982043 (chr6:32164665), intronic (*NOTCH4*) | *AGER* | CT | C | 0.047 | 0.99 | 1.081 | 0.058 | 1.8x${10}^{-1}$ | NO |
| rs146088795 (chr6:142640832), intronic (*GPR126*) | *GPR126* | A | G | 0.016 | 0.987 | 0.934 | 0.104 | 5.1x${10}^{-1}$ | YES |
| rs7174934 (chr15:71571345), intronic (*THSD4*) | *THSD4* | G | A | 0.414 | 0.987 | 1.002 | 0.026 | 9.39x${10}^{-1}$ | NO |
| rs75958385 (chr16:75403497), intronic (*CFDP1*) | *CFDP1* | G | A | 0.029 | 0.956 | 1.185 | 0.075 | 2.4x${10}^{-2}$ | NO |
| rs199588075 (chr21:35679578), transcript (*AP000318.2*) | *KCNE2* | CT | C | 0.031 | 0.955 | 0.886 | 0.079 | 1.25x${10}^{-1}$ | NO |
