# Supplementary material for: Targeted Sequencing of Lung Function Loci in Chronic Obstructive Pulmonary Disease Cases and Controls
Source: PLoS One. 2017 Jan 23;12(1):e0170222. doi: 10.1371/journal.pone.0170222 (PMC5256917; doi:10.1371/journal.pone.0170222)
Supplement: S5 Table — The column “GWAS gene” presents the gene reported in the lung function GWAS [9] for each region. Abbreviations: chr = chromosome, Ref = reference, Alt = alternative, MAF = minor allele frequency, OR = odds ratio, SE = standard error. (DOCX) [file pone.0170222.s008.docx]

S5 Table Conditional analysis in *HTR4* region

The column “GWAS gene” presents the gene reported in the lung function GWAS [[5](#_ENREF_5)] for each region. Abbreviations: chr=chromosome, Ref=reference, Alt=alternative, MAF=minor allele frequency, OR=odds ratio, SE=standard error.

| **rs number (chr: position), function** | **GWAS gene** | **Ref allele** | **Alt allele** | **MAF** | **Imputation information** | **Unconditional analysis** | | | **Joint analysis** | | | **r** |
| --- | --- | --- | --- | --- | --- | --- | --- | --- | --- | --- | --- | --- |
|  |  |  |  |  |  | **OR** | **SE** | **P-value** | **OR** | **SE** | **P-value** |  |
| rs999741 (chr5:147727048), transcript (*RP11-373N22.3*) | *HTR4* | C | G | 0.256 | 0.999 | 0.915 | 0.029 | 2.35x${10}^{-3}$ | 0.969 | 0.033 | 3.3x${10}^{-1}$ | 0.45 |
| rs1985524 (chr5:147847788), intronic (*HTR4*) | *HTR4* | G | C | 0.445 | 1 | 0.882 | 0.026 | 1.08x${10}^{-6}$ | 0.893 | 0.029 | 8.54x${10}^{-5}$ |  |
