# Supplementary material for: Targeted Sequencing of Lung Function Loci in Chronic Obstructive Pulmonary Disease Cases and Controls
Source: PLoS One. 2017 Jan 23;12(1):e0170222. doi: 10.1371/journal.pone.0170222 (PMC5256917; doi:10.1371/journal.pone.0170222)
Supplement: S6 Table — Results of COPD risk associations for variants previously associated with lung function [7–10] are presented here ordered by chromosome and position and by P-value significance for each variant. P-values < 0.05 are highlighted in bold. “GWAS gene” presents the closest gene to the lung function sentinel SNP reported in [9]. Abbreviations: MAF = minor allele frequency, N alt ac = number of alternative allele counts, N ref ac = number of reference allele counts, freq = frequency, OR = odds ratio. (DOCX) [file pone.0170222.s009.docx]

S6 Table Single variant results for known variants

Results of COPD risk associations for variants previously associated with lung function [[5-8](#_ENREF_5)] are presented here ordered by chromosome and position and by P-value significance for each variant. P-values < 0.05 are highlighted in bold. “GWAS gene” presents the closest gene to the lung function sentinel SNP reported in [[5](#_ENREF_5)]. Abbreviations: MAF=minor allele frequency, N alt ac=number of alternative allele counts, N ref ac=number of reference allele counts, freq=frequency, OR=odds ratio.

| **rs number (chr: position)** | **GWAS gene** | **Calling algorithm** | **Ref allele** | **Alt allele** | **MAF** | **Alt allele freq in cases** | **Alt allele freq in controls** | **OR** | **P-value** | **Consistent direction of effect?** |
| --- | --- | --- | --- | --- | --- | --- | --- | --- | --- | --- |
| rs2284746 (chr1:17306675) | *MFAP2* | vipR | G | C | 0.48 | 0.492 | 0.460 | 1.14 | 3.3x${10}^{-1}$ | NO |
|  |  | syzygy | C | G | 0.46 | 0.547 | 0.532 | 1.06 | 6.28x${10}^{-1}$ | YES |
|  |  | SNVer | C | G | 0.48 | 0.530 | 0.516 | 1.06 | 6.71x${10}^{-1}$ | YES |
| rs993925 (chr1:218860068) | *TGFB2* | SNVer | C | T | 0.38 | 0.395 | 0.358 | 1.17 | 2.12x${10}^{-1}$ | NO |
|  |  | syzygy | C | T | 0.38 | 0.398 | 0.364 | 1.16 | 2.62x${10}^{-1}$ | NO |
|  |  | vipR | C | T | 0.39 | 0.400 | 0.373 | 1.12 | 4.19x${10}^{-1}$ | NO |
| rs2571445 (chr2:218683154) | *TNS1* | SNVer | A | G | 0.49 | 0.500 | 0.536 | 0.87 | 2.5x${10}^{-1}$ | YES |
|  |  | vipR | G | A | 0.44 | 0.590 | 0.520 | 1.33 | 2.68x${10}^{-1}$ | YES |
|  |  | syzygy | A | G | 0.48 | 0.507 | 0.532 | 0.90 | 4.31x${10}^{-1}$ | YES |
| rs12477314 (chr2:239877148) | *HDAC4* | SNVer | C | T | 0.18 | 0.190 | 0.172 | 1.13 | 4.8x${10}^{-1}$ | NO |
|  |  | syzygy | C | T | 0.18 | 0.190 | 0.176 | 1.10 | 5.84x${10}^{-1}$ | NO |
|  |  | vipR | C | T | 0.21 | 0.218 | 0.200 | 1.11 | 6x${10}^{-1}$ | NO |
| rs1529672 (chr3:25520582) | *RARB* | SNVer | C | A | 0.15 | 0.143 | 0.166 | 0.84 | 3.14x${10}^{-1}$ | YES |
|  |  | syzygy | C | A | 0.16 | 0.148 | 0.168 | 0.86 | 4.06x${10}^{-1}$ | YES |
|  |  | vipR | C | A | 0.16 | 0.155 | 0.164 | 0.93 | 7.36x${10}^{-1}$ | YES |
| rs1344555 (chr3:169300219) | *MECOM* | SNVer | C | T | 0.2 | 0.238 | 0.154 | 1.72 | **4.93x**$\boldsymbol{10}^{\boldsymbol{-4}}$ | YES |
|  |  | syzygy | C | T | 0.21 | 0.247 | 0.162 | 1.69 | **5.93x**$\boldsymbol{10}^{\boldsymbol{-4}}$ | YES |
|  |  | vipR | C | T | 0.23 | 0.260 | 0.180 | 1.60 | **9.29x**$\boldsymbol{10}^{\boldsymbol{-3}}$ | YES |
| rs2045517 (chr4:89870964) | *FAM13A* | vipR | C | T | 0.45 | 0.456 | 0.444 | 1.05 | 7.44x${10}^{-1}$ | YES |
|  |  | SNVer | C | T | 0.43 | 0.433 | 0.438 | 0.98 | 9.03x${10}^{-1}$ | NO |
|  |  | syzygy | C | T | 0.44 | 0.440 | 0.438 | 1.01 | 9.51x${10}^{-1}$ | YES |
| rs10516526 (chr4:106688904) | *GSTCD* | vipR | A | G | 0.09 | 0.106 | 0.074 | 1.47 | 1.86x${10}^{-1}$ | NO |
|  |  | SNVer | A | G | 0.07 | 0.070 | 0.060 | 1.18 | 5.42x${10}^{-1}$ | NO |
|  |  | syzygy | A | G | 0.07 | 0.073 | 0.064 | 1.16 | 5.54x${10}^{-1}$ | NO |
| rs11100860 (chr4:145479139) | *HHIP* | SNVer | A | G | 0.37 | 0.337 | 0.410 | 0.73 | **1.44x**$\boldsymbol{10}^{\boldsymbol{-2}}$ | YES |
|  |  | syzygy | A | G | 0.37 | 0.340 | 0.404 | 0.76 | **3.28x**$\boldsymbol{10}^{\boldsymbol{-2}}$ | YES |
|  |  | vipR | A | G | 0.4 | 0.398 | 0.406 | 0.97 | 8.37x${10}^{-1}$ | YES |
| rs153916 (chr5:95036700) | *SPATA9* | SNVer | C | T | 0.43 | 0.600 | 0.530 | 1.33 | **2.03x**$\boldsymbol{10}^{\boldsymbol{-2}}$ | YES |
|  |  | syzygy | C | T | 0.43 | 0.605 | 0.536 | 1.33 | **2.35x**$\boldsymbol{10}^{\boldsymbol{-2}}$ | YES |
|  |  | vipR | T | C | 0.45 | 0.424 | 0.474 | 0.82 | 1.33x${10}^{-1}$ | YES |
| rs1985524 (chr5:147847788) | *HTR4* | SNVer | G | C | 0.41 | 0.378 | 0.448 | 0.75 | **2.27x**$\boldsymbol{10}^{\boldsymbol{-2}}$ | YES |
|  |  | syzygy | G | C | 0.41 | 0.383 | 0.446 | 0.77 | **3.67x**$\boldsymbol{10}^{\boldsymbol{-2}}$ | YES |
|  |  | vipR | G | C | 0.44 | 0.428 | 0.446 | 0.93 | 5.89x${10}^{-1}$ | YES |
| rs11134779 (chr5:156936766) | *ADAM19* | vipR | A | G | 0.35 | 0.332 | 0.375 | 0.83 | 1.83x${10}^{-1}$ | NO |
|  |  | SNVer | A | G | 0.32 | 0.312 | 0.342 | 0.87 | 3.01x${10}^{-1}$ | NO |
|  |  | syzygy | A | G | 0.33 | 0.317 | 0.338 | 0.91 | 4.78x${10}^{-1}$ | NO |
| rs6903823 (chr6:28322296) | *ZKSCAN3* | vipR | A | G | 0.25 | 0.225 | 0.294 | 0.70 | **1.98x**$\boldsymbol{10}^{\boldsymbol{-2}}$ | NO |
|  |  | syzygy | A | G | 0.23 | 0.220 | 0.234 | 0.92 | 6.13x${10}^{-1}$ | NO |
|  |  | SNVer | A | G | 0.24 | 0.240 | 0.244 | 0.98 | 9.41x${10}^{-1}$ | NO |
| rs2857595 (chr6:31568469) | *NCR3* | vipR | G | A | 0.24 | 0.253 | 0.227 | 1.16 | 3.91x${10}^{-1}$ | YES |
|  |  | SNVer | G | A | 0.22 | 0.222 | 0.218 | 1.02 | 9.42x${10}^{-1}$ | YES |
|  |  | syzygy | G | A | 0.22 | 0.222 | 0.224 | 0.99 | 9.42x${10}^{-1}$ | NO |
| rs2070600 (chr6:32151443) | *AGER* | SNVer | C | T | 0.06 | 0.074 | 0.053 | 1.42 | 2.33x${10}^{-1}$ | NO |
|  |  | syzygy | C | T | 0.07 | 0.080 | 0.062 | 1.32 | 2.91x${10}^{-1}$ | NO |
|  |  | vipR | C | T | 0.09 | 0.089 | 0.092 | 0.96 | 8.86x${10}^{-1}$ | YES |
| rs2798641 (chr6:109268050) | *ARMC2* | vipR | C | T | 0.19 | 0.175 | 0.209 | 0.80 | 1.7x${10}^{-1}$ | NO |
|  |  | SNVer | C | T | 0.18 | 0.167 | 0.198 | 0.81 | 1.82x${10}^{-1}$ | NO |
|  |  | syzygy | C | T | 0.18 | 0.172 | 0.192 | 0.87 | 3.88x${10}^{-1}$ | NO |
| rs262129 (chr6:142853144) | *LOC153910* | SNVer | A | G | 0.29 | 0.295 | 0.288 | 1.03 | 8.42x${10}^{-1}$ | NO |
|  |  | vipR | A | G | 0.31 | 0.307 | 0.311 | 0.98 | 9.45x${10}^{-1}$ | YES |
|  |  | syzygy | A | G | 0.29 | 0.292 | 0.292 | 1.00 | 1 | YES |
| rs16909859 (chr9:98204792) | *PTCH1* | SNVer | G | A | 0.05 | 0.042 | 0.064 | 0.64 | 1.03x${10}^{-1}$ | NO |
|  |  | syzygy | G | A | 0.05 | 0.047 | 0.062 | 0.74 | 2.84x${10}^{-1}$ | NO |
|  |  | vipR | G | A | 0.06 | 0.053 | 0.074 | 0.70 | 3.38x${10}^{-1}$ | NO |
| rs7068966 (chr10:12277992) | *CDC123* | vipR | C | T | 0.48 | 0.518 | 0.511 | 1.03 | 8.46x${10}^{-1}$ | NO |
|  |  | SNVer | C | T | 0.5 | 0.497 | 0.504 | 0.97 | 8.56x${10}^{-1}$ | YES |
|  |  | syzygy | C | T | 0.5 | 0.503 | 0.500 | 1.01 | 9.52x${10}^{-1}$ | NO |
| rs11001819 (chr10:78315224) | *C10orf11* | vipR | G | A | 0.48 | 0.495 | 0.462 | 1.14 | 3.4x${10}^{-1}$ | NO |
|  |  | syzygy | G | A | 0.47 | 0.485 | 0.456 | 1.12 | 3.63x${10}^{-1}$ | NO |
|  |  | SNVer | G | A | 0.46 | 0.472 | 0.450 | 1.09 | 5.04x${10}^{-1}$ | NO |
| rs11172113 (chr12:57527283) | *LRP1* | vipR | T | C | 0.44 | 0.460 | 0.423 | 1.16 | 3.61x${10}^{-1}$ | NO |
|  |  | SNVer | T | C | 0.4 | 0.388 | 0.408 | 0.92 | 5.36x${10}^{-1}$ | YES |
|  |  | syzygy | T | C | 0.4 | 0.390 | 0.410 | 0.92 | 5.36x${10}^{-1}$ | YES |
| rs1036429 (chr12:96271428) | *CCDC38* | vipR | C | T | 0.25 | 0.240 | 0.254 | 0.93 | 6.72x${10}^{-1}$ | YES |
|  |  | syzygy | T | C | 0.22 | 0.778 | 0.772 | 1.04 | 8.28x${10}^{-1}$ | YES |
|  |  | SNVer | T | C | 0.23 | 0.767 | 0.766 | 1.00 | 1 | YES |
| rs8033889 (chr15:71680080) | *THSD4* | SNVer | G | T | 0.2 | 0.193 | 0.214 | 0.88 | 4.08x${10}^{-1}$ | NO |
|  |  | syzygy | G | T | 0.21 | 0.200 | 0.218 | 0.90 | 5.02x${10}^{-1}$ | NO |
|  |  | vipR | G | T | 0.21 | 0.210 | 0.210 | 1.00 | 1 | - |
| rs12447804 (chr16:58075282) | *MMP15* | vipR | C | T | 0.22 | 0.222 | 0.214 | 1.05 | 7.97x${10}^{-1}$ | YES |
|  |  | SNVer | C | T | 0.2 | 0.198 | 0.202 | 0.98 | 8.8x${10}^{-1}$ | NO |
|  |  | syzygy | C | T | 0.2 | 0.200 | 0.200 | 1.00 | 1 | - |
| rs35263058 (chr16:75391937) | *CFDP1* | SNVer | T | C | 0.41 | 0.580 | 0.608 | 0.89 | 3.56x${10}^{-1}$ | NO |
|  |  | syzygy | T | C | 0.41 | 0.587 | 0.604 | 0.93 | 5.79x${10}^{-1}$ | NO |
|  |  | vipR | C | T | 0.44 | 0.442 | 0.425 | 1.07 | 6.36x${10}^{-1}$ | NO |
| rs9978142 (chr21:35652239) | *KCNE2* | SNVer | A | T | 0.16 | 0.167 | 0.152 | 1.12 | 5.63x${10}^{-1}$ | YES |
|  |  | vipR | A | T | 0.17 | 0.165 | 0.171 | 0.96 | 8.03x${10}^{-1}$ | NO |
|  |  | syzygy | A | T | 0.16 | 0.163 | 0.158 | 1.04 | 8.69x${10}^{-1}$ | YES |
