# Supplementary material for: Targeted Sequencing of Lung Function Loci in Chronic Obstructive Pulmonary Disease Cases and Controls
Source: PLoS One. 2017 Jan 23;12(1):e0170222. doi: 10.1371/journal.pone.0170222 (PMC5256917; doi:10.1371/journal.pone.0170222)
Supplement: S7 Table — Results are presented for loci (sliding windows or genes) that reach the threshold for follow-up after sensitivity analyses either with (“Independent variants and variants not in UK10K+1000G”) or without (“Independent variants”) including variants not in joint 1000 Genomes Project [20] and UK10K [21] reference panel. Abbreviations: N = number of variants, P = P-value, UK10K+1000G = joint 1000 Genomes Project and UK10K reference panel. a) Burden test results in stage 1b) C-alpha test results in stage 1 i) Sliding windowii) Gene basediii) Exon based (DOCX) [file pone.0170222.s010.docx]

S7 Table Collapsing methods stage 1 results meeting the significance threshold

Results are presented for loci (sliding windows or genes) that reach the threshold for follow-up after sensitivity analyses either with (“Independent variants and variants not in UK10K+1000G”) or without (“Independent variants”) including variants not in joint 1000 Genomes Project [[2](#_ENREF_2)] and UK10K [[4](#_ENREF_4)] reference panel. Abbreviations: N=number of variants, P=P-value, UK10K+1000G=joint 1000 Genomes Project and UK10K reference panel.

1. Burden test results in stage 1

| **Locus** | **Threshold** | **All variants** | | | | | | **Independent variants** | | | | | | **Independent variants and variants not in UK10K+1000G** | | | | | |
| --- | --- | --- | --- | --- | --- | --- | --- | --- | --- | --- | --- | --- | --- | --- | --- | --- | --- | --- | --- |
|  |  | **vipR** | | **SNVer** | | **Syzygy** | | **vipR** | | **SNVer** | | **Syzygy** | | **vipR** | | **SNVer** | | **Syzygy** | |
|  |  | P | N | P | N | P | N | P | N | P | N | P | N | P | N | P | N | P | N |
| chr3:168984786-168987786 | 1.87x${10}^{-4}$ | - | - | 2.25x${10}^{-4}$ | 5 | 7.87x${10}^{-5}$ | 5 | - | - | 7.24x${10}^{-3}$ | 2 | 9.81x${10}^{-4}$ | 2 | - | - | 2.25x${10}^{-4}$ | 5 | 7.87x${10}^{-5}$ | 5 |
| *FLJ20184* | 1x${10}^{-2}$ | 1 | 27 | 1.28x${10}^{-2}$ | 42 | 5.12x${10}^{-4}$ | 46 | 1 | 15 | 1.43x${10}^{-2}$ | 23 | 1.12x${10}^{-4}$ | 25 | 1 | 22 | 4.21x${10}^{-2}$ | 37 | 1.57x${10}^{-3}$ | 41 |
| chr4:145278600-145281600 | 2.76x${10}^{-4}$ | - | - | 2.34x${10}^{-4}$ | 4 | 2.15x${10}^{-4}$ | 4 | - | - | 3.76x${10}^{-3}$ | 2 | 1.38x${10}^{-3}$ | 2 | - | - | 2.34x${10}^{-4}$ | 4 | 2.15x${10}^{-4}$ | 4 |
| *ITK* | 8.33x${10}^{-3}$ | 1 | 13 | 3.98x${10}^{-4}$ | 28 | 1.41x${10}^{-4}$ | 29 | 1 | 10 | 5.81x${10}^{-5}$ | 20 | 5.01x${10}^{-5}$ | 21 | 1 | 12 | 3.98x${10}^{-4}$ | 28 | 1.64x${10}^{-4}$ | 28 |
| *GPR126* | 2.5x${10}^{-2}$ | 7.18x${10}^{-1}$ | 63 | 2.04x${10}^{-3}$ | 110 | 3.21x${10}^{-2}$ | 109 | 7.98x${10}^{-1}$ | 33 | 8.11x${10}^{-2}$ | 61 | 3.91x${10}^{-1}$ | 60 | 5.15x${10}^{-1}$ | 50 | 1.51x${10}^{-3}$ | 93 | 4.65x${10}^{-2}$ | 91 |

1. C-alpha test results in stage 1
2. Sliding window

| **Locus** | **Threshold** | **All variants** | | | | | | **Independent variants** | | | | | | **Independent variants and variants**  **not in UK10K+1000G** | | | | | |
| --- | --- | --- | --- | --- | --- | --- | --- | --- | --- | --- | --- | --- | --- | --- | --- | --- | --- | --- | --- |
|  |  | **vipR** | | **SNVer** | | **Syzygy** | | **vipR** | | **SNVer** | | **Syzygy** | | **vipR** | | **SNVer** | | **Syzygy** | |
|  |  | **N** | **P** | **N** | **P** | **N** | **P** | **N** | **P** | **N** | **P** | **N** | **P** | **N** | **P** | **N** | **P** | **N** | **P** |
| chr1:218531175-218534175 | 4.24x${10}^{-4}$ | 1 | - | 2 | 7.67x${10}^{-7}$ | 2 | 7.67x${10}^{-7}$ | 1 | - | 2 | 7.67x${10}^{-7}$ | 2 | 7.67x${10}^{-7}$ | 1 | - | 2 | 7.67x${10}^{-7}$ | 2 | 7.67x${10}^{-7}$ |
| chr2:218807794-218810794 | 1.09x${10}^{-3}$ | 3 | 6.33x${10}^{-5}$ | 4 | 1.91x${10}^{-5}$ | 3 | 2.92x${10}^{-3}$ | 2 | 9.25x${10}^{-4}$ | 3 | 1.19x${10}^{-4}$ | 2 | 3.5x${10}^{-2}$ | 2 | 9.25x${10}^{-4}$ | 3 | 1.19x${10}^{-4}$ | 2 | 3.5x${10}^{-2}$ |
| chr2:239890616-239893616 | 3.68x${10}^{-4}$ | 1 | - | 3 | 5.76x${10}^{-6}$ | 3 | 6.33x${10}^{-5}$ | - | - | 2 | 6.05x${10}^{-6}$ | 2 | 6.65x${10}^{-5}$ | 1 | - | 3 | 5.76x${10}^{-6}$ | 3 | 6.33x${10}^{-5}$ |
| chr2:239971616-239974616 | 3.68x${10}^{-4}$ | 1 | - | 3 | 8.03x${10}^{-11}$ | 3 | 1.33x${10}^{-9}$ | 1 | - | 3 | 8.03x${10}^{-11}$ | 3 | 1.33x${10}^{-9}$ | 1 | - | 3 | 8.03x${10}^{-11}$ | 3 | 1.33x${10}^{-9}$ |
| chr2:239973116-239976116 | 3.68x${10}^{-4}$ | 1 | - | 4 | 2.71x${10}^{-13}$ | 4 | 3.4x${10}^{-12}$ | 1 | - | 3 | 8.03x${10}^{-11}$ | 3 | 1.33x${10}^{-9}$ | 1 | - | 3 | 8.03x${10}^{-11}$ | 3 | 1.33x${10}^{-9}$ |
| chr2:240325616-240328616 | 3.68x${10}^{-4}$ | 2 | 2.61x${10}^{-6}$ | 2 | 3.95x${10}^{-11}$ | 2 | 7.05x${10}^{-3}$ | 2 | 2.61x${10}^{-6}$ | 2 | 3.95x${10}^{-11}$ | 2 | 7.05x${10}^{-3}$ | 2 | 2.61x${10}^{-6}$ | 2 | 3.95x${10}^{-11}$ | 2 | 7.05x${10}^{-3}$ |
| chr3:168984786-168987786 | 1.87x${10}^{-4}$ | 2 | 2.48x${10}^{-2}$ | 5 | 1.5x${10}^{-5}$ | 5 | 2.74x${10}^{-6}$ | 1 | - | 2 | 1.12x${10}^{-4}$ | 2 | 1.06x${10}^{-5}$ | 2 | 2.48x${10}^{-2}$ | 5 | 1.5x${10}^{-5}$ | 5 | 2.74x${10}^{-6}$ |
| chr3:169238286-169241286 | 1.87x${10}^{-4}$ | 1 | - | 4 | 4.52x${10}^{-5}$ | 4 | 5.34x${10}^{-6}$ | 1 | - | 2 | 3.5x${10}^{-4}$ | 2 | 1.46x${10}^{-5}$ | 1 | - | 4 | 4.52x${10}^{-5}$ | 4 | 5.34x${10}^{-6}$ |
| chr3:169310286-169313286 | 1.87x${10}^{-4}$ | 2 | 2.48x${10}^{-2}$ | 3 | 1.99x${10}^{-8}$ | 3 | 1.99x${10}^{-8}$ | 2 | 2.48x${10}^{-2}$ | 2 | 6.65x${10}^{-5}$ | 2 | 2.85x${10}^{-6}$ | 2 | 2.48x${10}^{-2}$ | 2 | 6.65x${10}^{-5}$ | 2 | 2.85x${10}^{-6}$ |
| chr3:169311786-169314786 | 1.87x${10}^{-4}$ | 2 | 2.48x${10}^{-2}$ | 3 | 1.99x${10}^{-8}$ | 3 | 1.99x${10}^{-8}$ | 2 | 2.48x${10}^{-2}$ | 2 | 6.65x${10}^{-5}$ | 2 | 2.85x${10}^{-6}$ | 2 | 2.48x${10}^{-2}$ | 2 | 6.65x${10}^{-5}$ | 2 | 2.85x${10}^{-6}$ |
| chr3:169340286-169343286 | 1.87x${10}^{-4}$ | 3 | 2.95x${10}^{-9}$ | 4 | 5.29x${10}^{-6}$ | 4 | 8.75x${10}^{-6}$ | 2 | 7.82x${10}^{-9}$ | 2 | 7.06x${10}^{-5}$ | 2 | 7.06x${10}^{-5}$ | 2 | 7.82x${10}^{-9}$ | 3 | 3.82x${10}^{-5}$ | 3 | 6.7x${10}^{-5}$ |
| chr3:169341786-169344786 | 1.87x${10}^{-4}$ | 6 | 2.54x${10}^{-10}$ | 5 | 3.03x${10}^{-6}$ | 6 | 1.37x${10}^{-10}$ | 4 | 1.7x${10}^{-9}$ | 2 | 7.06x${10}^{-5}$ | 3 | 9.65x${10}^{-10}$ | 5 | 6.58x${10}^{-10}$ | 4 | 2.05x${10}^{-5}$ | 5 | 4.54x${10}^{-10}$ |
| chr3:169371786-169374786 | 1.87x${10}^{-4}$ | 2 | 7.36x${10}^{-2}$ | 7 | 2.69x${10}^{-5}$ | 6 | 1.36x${10}^{-5}$ | 1 | - | 4 | 2.18x${10}^{-4}$ | 3 | 6.3x${10}^{-5}$ | 2 | 7.36x${10}^{-2}$ | 7 | 2.69x${10}^{-5}$ | 6 | 1.36x${10}^{-5}$ |
| chr3:169373286-169376286 | 1.87x${10}^{-4}$ | 4 | 4.59x${10}^{-2}$ | 6 | 2.64x${10}^{-7}$ | 6 | 9.73x${10}^{-7}$ | 3 | 1.26x${10}^{-1}$ | 3 | 5.39x${10}^{-6}$ | 3 | 5.39x${10}^{-6}$ | 4 | 4.59x${10}^{-2}$ | 6 | 2.64x${10}^{-7}$ | 6 | 9.73x${10}^{-7}$ |
| chr3:25464333-25467333 | 5.81x${10}^{-4}$ | 3 | 1.25x${10}^{-4}$ | 4 | 1.63x${10}^{-5}$ | 5 | 9.01x${10}^{-3}$ | 3 | 1.25x${10}^{-4}$ | 3 | 1.51x${10}^{-5}$ | 4 | 5.69x${10}^{-3}$ | 3 | 1.25x${10}^{-4}$ | 4 | 1.63x${10}^{-5}$ | 5 | 9.01x${10}^{-3}$ |
| chr3:25510833-25513833 | 5.81x${10}^{-4}$ | 4 | 1.62x${10}^{-3}$ | 3 | 5.19x${10}^{-8}$ | 2 | 5.02x${10}^{-7}$ | 4 | 1.62x${10}^{-3}$ | 3 | 5.19x${10}^{-8}$ | 2 | 5.02x${10}^{-7}$ | 4 | 1.62x${10}^{-3}$ | 3 | 5.19x${10}^{-8}$ | 2 | 5.02x${10}^{-7}$ |
| chr3:25512333-25515333 | 5.81x${10}^{-4}$ | 3 | 2.03x${10}^{-3}$ | 2 | 6x${10}^{-8}$ | 2 | 5.02x${10}^{-7}$ | 3 | 2.03x${10}^{-3}$ | 2 | 6x${10}^{-8}$ | 2 | 5.02x${10}^{-7}$ | 3 | 2.03x${10}^{-3}$ | 2 | 6x${10}^{-8}$ | 2 | 5.02x${10}^{-7}$ |
| chr3:25527333-25530333 | 5.81x${10}^{-4}$ | 1 | - | 2 | 6.65x${10}^{-5}$ | 2 | 6.65x${10}^{-5}$ | 1 | - | 2 | 6.65x${10}^{-5}$ | 2 | 6.65x${10}^{-5}$ | 1 | - | 2 | 6.65x${10}^{-5}$ | 2 | 6.65x${10}^{-5}$ |
| chr3:25599333-25602333 | 5.81x${10}^{-4}$ | 2 | 3.76x${10}^{-5}$ | 2 | 1.52x${10}^{-4}$ | 2 | 1.52x${10}^{-4}$ | 2 | 3.76x${10}^{-5}$ | 2 | 1.52x${10}^{-4}$ | 2 | 1.52x${10}^{-4}$ | 2 | 3.76x${10}^{-5}$ | 2 | 1.52x${10}^{-4}$ | 2 | 1.52x${10}^{-4}$ |
| chr3:25632333-25635333 | 5.81x${10}^{-4}$ | 2 | 2.48x${10}^{-2}$ | 6 | 1.14x${10}^{-5}$ | 6 | 7.35x${10}^{-4}$ | 2 | 2.48x${10}^{-2}$ | 6 | 1.14x${10}^{-5}$ | 6 | 7.35x${10}^{-4}$ | 2 | 2.48x${10}^{-2}$ | 6 | 1.14x${10}^{-5}$ | 6 | 7.35x${10}^{-4}$ |
| chr3:25633833-25636833 | 5.81x${10}^{-4}$ | 1 | - | 7 | 3.36x${10}^{-7}$ | 7 | 1.87x${10}^{-5}$ | 1 | - | 5 | 9.43x${10}^{-7}$ | 5 | 4.73x${10}^{-5}$ | 1 | - | 7 | 3.36x${10}^{-7}$ | 7 | 1.87x${10}^{-5}$ |
| chr4:106514233-106517233 | 2.69x${10}^{-4}$ | 1 | - | 2 | 3.12x${10}^{-9}$ | 2 | 7.37x${10}^{-9}$ | 1 | - | 2 | 3.12x${10}^{-9}$ | 2 | 7.37x${10}^{-9}$ | 1 | - | 2 | 3.12x${10}^{-9}$ | 2 | 7.37x${10}^{-9}$ |
| chr4:106515733-106518733 | 2.69x${10}^{-4}$ | 2 | 4.19x${10}^{-3}$ | 5 | 3.15x${10}^{-9}$ | 5 | 8.26x${10}^{-9}$ | 1 | - | 3 | 2.17x${10}^{-8}$ | 3 | 5.09x${10}^{-8}$ | 2 | 4.19x${10}^{-3}$ | 5 | 3.15x${10}^{-9}$ | 5 | 8.26x${10}^{-9}$ |
| chr4:145265100-145268100 | 2.76x${10}^{-4}$ | 3 | 5.88x${10}^{-1}$ | 6 | 4.45x${10}^{-7}$ | 7 | 1.26x${10}^{-11}$ | 3 | 5.88x${10}^{-1}$ | 3 | 1.71x${10}^{-4}$ | 4 | 1.76x${10}^{-8}$ | 3 | 5.88x${10}^{-1}$ | 5 | 6.4x${10}^{-5}$ | 6 | 1.66x${10}^{-9}$ |
| chr4:145266600-145269600 | 2.76x${10}^{-4}$ | 5 | 5.57x${10}^{-3}$ | 9 | 1.04x${10}^{-8}$ | 8 | 6.12x${10}^{-11}$ | 3 | 1.73x${10}^{-2}$ | 4 | 1.44x${10}^{-5}$ | 4 | 2.66x${10}^{-7}$ | 5 | 5.57x${10}^{-3}$ | 8 | 1.33x${10}^{-6}$ | 7 | 7.91x${10}^{-9}$ |
| chr4:145268100-145271100 | 2.76x${10}^{-4}$ | 8 | 1.45x${10}^{-3}$ | 9 | 9.28x${10}^{-7}$ | 8 | 5.78x${10}^{-8}$ | 5 | 5.57x${10}^{-3}$ | 5 | 6.14x${10}^{-6}$ | 5 | 2.39x${10}^{-7}$ | 8 | 1.45x${10}^{-3}$ | 9 | 9.28x${10}^{-7}$ | 8 | 5.78x${10}^{-8}$ |
| chr4:145269600-145272600 | 2.76x${10}^{-4}$ | 4 | 4.59x${10}^{-2}$ | 4 | 1.37x${10}^{-4}$ | 4 | 4.21x${10}^{-5}$ | 3 | 5.88x${10}^{-2}$ | 3 | 1.71x${10}^{-4}$ | 3 | 3.89x${10}^{-5}$ | 4 | 4.59x${10}^{-2}$ | 4 | 1.37x${10}^{-4}$ | 4 | 4.21x${10}^{-5}$ |
| chr4:145272600-145275600 | 2.76x${10}^{-4}$ | 3 | 1.01x${10}^{-2}$ | 4 | 8.26x${10}^{-6}$ | 4 | 3.01x${10}^{-6}$ | 3 | 1.01x${10}^{-2}$ | 4 | 8.26x${10}^{-6}$ | 4 | 3.01x${10}^{-6}$ | 3 | 1.01x${10}^{-2}$ | 4 | 8.26x${10}^{-6}$ | 4 | 3.01x${10}^{-6}$ |
| chr4:145278600-145281600 | 2.76x${10}^{-4}$ | 3 | 1.26x${10}^{-1}$ | 4 | 2.55x${10}^{-4}$ | 4 | 1.46x${10}^{-5}$ | 2 | 1.75x${10}^{-1}$ | 2 | 4.02x${10}^{-4}$ | 2 | 1.69x${10}^{-5}$ | 3 | 1.26x${10}^{-1}$ | 4 | 2.55x${10}^{-4}$ | 4 | 1.46x${10}^{-5}$ |
| chr4:145289100-145292100 | 2.76x${10}^{-4}$ | 4 | 4.59x${10}^{-2}$ | 7 | 2.91x${10}^{-6}$ | 7 | 4.52x${10}^{-6}$ | 3 | 5.88x${10}^{-2}$ | 5 | 3.74x${10}^{-9}$ | 5 | 2.05x${10}^{-8}$ | 3 | 5.88x${10}^{-2}$ | 5 | 3.74x${10}^{-9}$ | 5 | 2.05x${10}^{-8}$ |
| chr4:145290600-145293600 | 2.76x${10}^{-4}$ | 2 | 1.75x${10}^{-1}$ | 5 | 2.66x${10}^{-7}$ | 5 | 1.28x${10}^{-5}$ | 1 | - | 3 | 6.81x${10}^{-7}$ | 3 | 3.33x${10}^{-5}$ | 2 | 1.75x${10}^{-1}$ | 4 | 6.31x${10}^{-7}$ | 4 | 3.06x${10}^{-5}$ |
| chr4:145293600-145296600 | 2.76x${10}^{-4}$ | 3 | 8.88x${10}^{-3}$ | 6 | 1.58x${10}^{-8}$ | 5 | 3.92x${10}^{-7}$ | 1 | - | 2 | 5.4x${10}^{-4}$ | 2 | 1.11x${10}^{-5}$ | 1 | - | 3 | 5.31x${10}^{-3}$ | 3 | 1.19x${10}^{-4}$ |
| chr4:145332600-145335600 | 2.76x${10}^{-4}$ | 5 | 8.25x${10}^{-3}$ | 6 | 1.14x${10}^{-9}$ | 7 | 3.66x${10}^{-13}$ | 2 | 3.47x${10}^{-2}$ | 3 | 1.84x${10}^{-5}$ | 4 | 7.88x${10}^{-7}$ | 2 | 3.47x${10}^{-2}$ | 3 | 1.84x${10}^{-5}$ | 4 | 7.88x${10}^{-7}$ |
| chr4:145334100-145337100 | 2.76x${10}^{-4}$ | 7 | 2.72x${10}^{-5}$ | 9 | 1.48x${10}^{-17}$ | 10 | 2.85x${10}^{-17}$ | 2 | 1.12x${10}^{-2}$ | 2 | 9.84x${10}^{-6}$ | 4 | 3.08x${10}^{-4}$ | 3 | 8.88x${10}^{-3}$ | 3 | 9.18x${10}^{-5}$ | 4 | 3.08x${10}^{-4}$ |
| chr4:145335600-145338600 | 2.76x${10}^{-4}$ | 10 | 1.72x${10}^{-7}$ | 13 | 1.25x${10}^{-27}$ | 13 | 2.12x${10}^{-26}$ | 2 | 3.61x${10}^{-3}$ | 2 | 1.09x${10}^{-7}$ | 3 | 3.62x${10}^{-5}$ | 3 | 2.85x${10}^{-3}$ | 4 | 7.58x${10}^{-7}$ | 4 | 3.77x${10}^{-5}$ |
| chr4:145341600-145344600 | 2.76x${10}^{-4}$ | 2 | 7.34x${10}^{-2}$ | 4 | 3.65x${10}^{-8}$ | 4 | 3.54x${10}^{-12}$ | 1 | - | 2 | 1.42x${10}^{-4}$ | 2 | 9.27x${10}^{-8}$ | 1 | - | 2 | 1.42x${10}^{-4}$ | 2 | 9.27x${10}^{-8}$ |
| chr4:145382100-145385100 | 2.76x${10}^{-4}$ | 5 | 2.78x${10}^{-3}$ | 6 | 3.33x${10}^{-9}$ | 6 | 4.3x${10}^{-10}$ | 4 | 7.04x${10}^{-3}$ | 5 | 4.57x${10}^{-7}$ | 5 | 5.79x${10}^{-8}$ | 4 | 7.04x${10}^{-3}$ | 5 | 4.57x${10}^{-7}$ | 5 | 5.79x${10}^{-8}$ |
| chr4:145383600-145386600 | 2.76x${10}^{-4}$ | 3 | 5.56x${10}^{-2}$ | 4 | 9.98x${10}^{-7}$ | 4 | 4.91x${10}^{-7}$ | 2 | 1.75x${10}^{-1}$ | 3 | 1.43x${10}^{-4}$ | 3 | 6.99x${10}^{-5}$ | 2 | 1.75x${10}^{-1}$ | 3 | 1.43x${10}^{-4}$ | 3 | 6.99x${10}^{-5}$ |
| chr4:89812605-89815605 | 5x${10}^{-4}$ | 2 | 7.36x${10}^{-2}$ | 2 | 9.25x${10}^{-4}$ | 2 | 3.76x${10}^{-5}$ | 2 | 7.36x${10}^{-2}$ | 2 | 9.25x${10}^{-4}$ | 2 | 3.76x${10}^{-5}$ | 2 | 7.36x${10}^{-2}$ | 2 | 9.25x${10}^{-4}$ | 2 | 3.76x${10}^{-5}$ |
| chr4:89814105-89817105 | 5x${10}^{-4}$ | 2 | 7.36x${10}^{-2}$ | 3 | 5.76x${10}^{-6}$ | 3 | 2.71x${10}^{-6}$ | 2 | 7.36x${10}^{-2}$ | 3 | 5.76x${10}^{-6}$ | 3 | 2.71x${10}^{-6}$ | 2 | 7.36x${10}^{-2}$ | 3 | 5.76x${10}^{-6}$ | 3 | 2.71x${10}^{-6}$ |
| chr5:147826118-147829118 | 4.9x${10}^{-4}$ | 2 | 3.76x${10}^{-5}$ | 2 | 9.87x${10}^{-14}$ | 2 | 4.7x${10}^{-6}$ | 2 | 3.76x${10}^{-5}$ | 2 | 9.87x${10}^{-14}$ | 2 | 4.7x${10}^{-6}$ | 2 | 3.76x${10}^{-5}$ | 2 | 9.87x${10}^{-14}$ | 2 | 4.7x${10}^{-6}$ |
| chr5:147829118-147832118 | 4.9x${10}^{-4}$ | - | - | 3 | 2.86x${10}^{-7}$ | 3 | 6.81x${10}^{-7}$ | - | - | 2 | 3.03x${10}^{-7}$ | 2 | 7.22x${10}^{-7}$ | - | - | 3 | 2.86x${10}^{-7}$ | 3 | 6.81x${10}^{-7}$ |
| chr5:147830618-147833618 | 4.9x${10}^{-4}$ | - | - | 3 | 2.86x${10}^{-7}$ | 3 | 6.81x${10}^{-7}$ | - | - | 2 | 3.03x${10}^{-7}$ | 2 | 7.22x${10}^{-7}$ | - | - | 3 | 2.86x${10}^{-7}$ | 3 | 6.81x${10}^{-7}$ |
| chr5:156912906-156915906 | 5.88x${10}^{-4}$ | 1 | - | 3 | 2.33x${10}^{-8}$ | 3 | 5.11x${10}^{-5}$ | 1 | - | 2 | 6x${10}^{-8}$ | 2 | 5.53x${10}^{-5}$ | 1 | - | 3 | 2.33x${10}^{-8}$ | 3 | 5.11x${10}^{-5}$ |
| chr9:98180197-98183197 | 9.8x${10}^{-4}$ | 1 | - | 3 | 1.88x${10}^{-6}$ | 3 | 1.06x${10}^{-4}$ | 1 | - | 2 | 2.68x${10}^{-7}$ | 2 | 3.5x${10}^{-5}$ | 1 | - | 3 | 1.88x${10}^{-6}$ | 3 | 1.06x${10}^{-4}$ |
| chr9:98181697-98184697 | 9.8x${10}^{-4}$ | 1 | - | 4 | 1.02x${10}^{-5}$ | 4 | 2.84x${10}^{-4}$ | 1 | - | 3 | 1.88x${10}^{-6}$ | 3 | 1.06x${10}^{-4}$ | 1 | - | 4 | 1.02x${10}^{-5}$ | 4 | 2.84x${10}^{-4}$ |
| chr10:12207674-12210674 | 1.09x${10}^{-3}$ | 2 | 2.7x${10}^{-2}$ | 4 | 9.11x${10}^{-5}$ | 4 | 1.6x${10}^{-7}$ | 2 | 2.7x${10}^{-2}$ | 3 | 4.8x${10}^{-4}$ | 3 | 1.02x${10}^{-6}$ | 2 | 2.7x${10}^{-2}$ | 4 | 9.11x${10}^{-5}$ | 4 | 1.6x${10}^{-7}$ |
| chr10:12209174-12212174 | 1.09x${10}^{-3}$ | 2 | 2.7x${10}^{-2}$ | 4 | 9.11x${10}^{-5}$ | 4 | 1.6x${10}^{-7}$ | 2 | 2.7x${10}^{-2}$ | 3 | 4.8x${10}^{-4}$ | 3 | 1.02x${10}^{-6}$ | 2 | 2.7x${10}^{-2}$ | 4 | 9.11x${10}^{-5}$ | 4 | 1.6x${10}^{-7}$ |
| chr10:77609018-77612018 | 1.71x${10}^{-4}$ | 3 | 1.55x${10}^{-1}$ | 5 | 2.17x${10}^{-4}$ | 5 | 6.3x${10}^{-5}$ | 3 | 1.55x${10}^{-1}$ | 4 | 2.04x${10}^{-4}$ | 4 | 5.91x${10}^{-5}$ | 3 | 1.55x${10}^{-1}$ | 5 | 2.17x${10}^{-4}$ | 5 | 6.3x${10}^{-5}$ |
| chr12:57529676-57532676 | 1.56x${10}^{-3}$ | 1 | - | 4 | 7.04x${10}^{-6}$ | 4 | 6.14x${10}^{-4}$ | 1 | - | 4 | 7.04x${10}^{-6}$ | 4 | 6.14x${10}^{-4}$ | 1 | - | 4 | 7.04x${10}^{-6}$ | 4 | 6.14x${10}^{-4}$ |
| chr12:96134582-96137582 | 5.05x${10}^{-4}$ | 4 | 4.66x${10}^{-3}$ | 3 | 4.44x${10}^{-6}$ | 2 | 5.33x${10}^{-4}$ | 3 | 1.02x${10}^{-2}$ | 2 | 9.28x${10}^{-6}$ | 2 | 5.33x${10}^{-4}$ | 4 | 4.66x${10}^{-3}$ | 3 | 4.44x${10}^{-6}$ | 2 | 5.33x${10}^{-4}$ |
| chr12:96136082-96139082 | 5.05x${10}^{-4}$ | 3 | 6.69x${10}^{-2}$ | 3 | 4.44x${10}^{-6}$ | 2 | 5.33x${10}^{-4}$ | 2 | 2.24x${10}^{-1}$ | 2 | 9.28x${10}^{-6}$ | 2 | 5.33x${10}^{-4}$ | 3 | 6.69x${10}^{-2}$ | 3 | 4.44x${10}^{-6}$ | 2 | 5.33x${10}^{-4}$ |
| chr12:96157082-96160082 | 5.05x${10}^{-4}$ | 1 | - | 2 | 4.69x${10}^{-7}$ | 2 | 4.84x${10}^{-9}$ | 1 | - | 2 | 4.69x${10}^{-7}$ | 2 | 4.84x${10}^{-9}$ | 1 | - | 2 | 4.69x${10}^{-7}$ | 2 | 4.84x${10}^{-9}$ |
| chr12:96158582-96161582 | 5.05x${10}^{-4}$ | 2 | 7.36x${10}^{-2}$ | 3 | 1.22x${10}^{-4}$ | 3 | 2.78x${10}^{-6}$ | 2 | 7.36x${10}^{-2}$ | 3 | 1.22x${10}^{-4}$ | 3 | 2.78x${10}^{-6}$ | 2 | 7.36x${10}^{-2}$ | 3 | 1.22x${10}^{-4}$ | 3 | 2.78x${10}^{-6}$ |
| chr12:96335582-96338582 | 5.05x${10}^{-4}$ | 1 | - | 3 | 2.56x${10}^{-9}$ | 3 | 5.15x${10}^{-5}$ | 1 | - | 3 | 2.56x${10}^{-9}$ | 3 | 5.15x${10}^{-5}$ | 1 | - | 3 | 2.56x${10}^{-9}$ | 3 | 5.15x${10}^{-5}$ |
| chr15:71704287-71707287 | 2.66x${10}^{-4}$ | 3 | 1.45x${10}^{-3}$ | 3 | 8.69x${10}^{-6}$ | 3 | 2.42x${10}^{-4}$ | 2 | 4.19x${10}^{-3}$ | 2 | 2.46x${10}^{-6}$ | 2 | 7.9x${10}^{-5}$ | 3 | 1.45x${10}^{-3}$ | 3 | 8.69x${10}^{-6}$ | 3 | 2.42x${10}^{-4}$ |
| chr16:58032243-58035243 | 1.61x${10}^{-3}$ | - | - | 3 | 1.67x${10}^{-7}$ | 4 | 2.68x${10}^{-6}$ | - | - | 2 | 3.99x${10}^{-9}$ | 2 | 3.25x${10}^{-4}$ | - | - | 2 | 3.99x${10}^{-9}$ | 3 | 1.81x${10}^{-5}$ |
| chr21:35645321-35648321 | 1.25x${10}^{-3}$ | 3 | 1.71x${10}^{-4}$ | 4 | 1.99x${10}^{-2}$ | 5 | 1.82x${10}^{-4}$ | 2 | 4.14x${10}^{-4}$ | 2 | 5.4x${10}^{-2}$ | 3 | 4.83x${10}^{-4}$ | 3 | 1.71x${10}^{-4}$ | 3 | 3.33x${10}^{-2}$ | 4 | 2.98x${10}^{-4}$ |
| chr21:35646821-35649821 | 1.25x${10}^{-3}$ | 4 | 1.59x${10}^{-6}$ | 5 | 1.65x${10}^{-2}$ | 5 | 1.82x${10}^{-4}$ | 2 | 4.14x${10}^{-4}$ | 2 | 5.4x${10}^{-2}$ | 3 | 4.83x${10}^{-4}$ | 3 | 1.71x${10}^{-4}$ | 3 | 3.33x${10}^{-2}$ | 4 | 2.98x${10}^{-4}$ |

1. Gene based

| **Locus** | **Threshold** | **All variants** | | | | | | **Independent variants** | | | | | | **Independent variants and variants**  **not in UK10K+1000G** | | | | | |
| --- | --- | --- | --- | --- | --- | --- | --- | --- | --- | --- | --- | --- | --- | --- | --- | --- | --- | --- | --- |
|  |  | **vipR** | | **SNVer** | | **Syzygy** | | **vipR** | | **SNVer** | | **Syzygy** | | **vipR** | | **SNVer** | | **Syzygy** | |
|  |  | **N** | **P** | **N** | **P** | **N** | **P** | **N** | **P** | **N** | **P** | **N** | **P** | **N** | **P** | **N** | **P** | **N** | **P** |
| *TGFB2* | 5x${10}^{-2}$ | 34 | 1.61x${10}^{-3}$ | 60 | 7.43x${10}^{-7}$ | 65 | 2.9x${10}^{-5}$ | 21 | 3.34x${10}^{-3}$ | 31 | 3.54x${10}^{-8}$ | 33 | 5.57x${10}^{-9}$ | 30 | 1.39x${10}^{-3}$ | 45 | 2.92x${10}^{-9}$ | 49 | 7.7x${10}^{-8}$ |
| *TNS1* | 5x${10}^{-2}$ | 22 | 5.32x${10}^{-4}$ | 69 | 2.07x${10}^{-2}$ | 72 | 3.74x${10}^{-1}$ | 12 | 1.52x${10}^{-2}$ | 41 | 3.38x${10}^{-2}$ | 42 | 3.08x${10}^{-1}$ | 19 | 5.75x${10}^{-3}$ | 61 | 5.2x${10}^{-3}$ | 62 | 1.56x${10}^{-1}$ |
| *HDAC4* | 5x${10}^{-2}$ | 65 | 3.43x${10}^{-34}$ | 174 | 1.42x${10}^{-22}$ | 189 | 2.95x${10}^{-4}$ | 32 | 3.2x${10}^{-24}$ | 93 | 2.07x${10}^{-18}$ | 100 | 2.15x${10}^{-5}$ | 41 | 7.87x${10}^{-26}$ | 141 | 8.81x${10}^{-25}$ | 151 | 2.63x${10}^{-5}$ |
| *RARB* | 2.5x${10}^{-2}$ | 68 | 1.48x${10}^{-17}$ | 125 | 7.24x${10}^{-12}$ | 127 | 2.44x${10}^{-7}$ | 48 | 4.33x${10}^{-13}$ | 72 | 1.17x${10}^{-7}$ | 76 | 5.33x${10}^{-4}$ | 60 | 1.02x${10}^{-15}$ | 111 | 6.04x${10}^{-11}$ | 112 | 3.14x${10}^{-8}$ |
| *MECOM* | 5x${10}^{-2}$ | 331 | 5.13x${10}^{-32}$ | 496 | 1.89x${10}^{-29}$ | 509 | 7.08x${10}^{-25}$ | 95 | 7.8x${10}^{-19}$ | 164 | 2.02x${10}^{-7}$ | 172 | 3.36x${10}^{-11}$ | 168 | 9.08x${10}^{-19}$ | 308 | 8.71x${10}^{-25}$ | 315 | 2.85x${10}^{-25}$ |
| *FAM13A* | 5x${10}^{-2}$ | 65 | 8.86x${10}^{-3}$ | 131 | 7.67x${10}^{-4}$ | 137 | 3.48x${10}^{-2}$ | 38 | 1.75x${10}^{-2}$ | 63 | 1.1x${10}^{-2}$ | 68 | 6.18x${10}^{-2}$ | 52 | 2.28x${10}^{-3}$ | 109 | 1.58x${10}^{-4}$ | 115 | 9.38x${10}^{-3}$ |
| *FLJ20184* | 1x${10}^{-2}$ | 27 | 5.52x${10}^{-11}$ | 42 | 7.52x${10}^{-7}$ | 46 | 1.93x${10}^{-3}$ | 15 | 1.82x${10}^{-8}$ | 23 | 9.67x${10}^{-6}$ | 25 | 1.24x${10}^{-3}$ | 22 | 1.65x${10}^{-11}$ | 37 | 6.27x${10}^{-7}$ | 41 | 2.37x${10}^{-3}$ |
| *HHIP* | 5x${10}^{-2}$ | 37 | 1.89x${10}^{-8}$ | 66 | 4.31x${10}^{-8}$ | 65 | 1.45x${10}^{-3}$ | 21 | 2.04x${10}^{-6}$ | 31 | 5.1x${10}^{-4}$ | 28 | 8.33x${10}^{-3}$ | 33 | 1.01x${10}^{-5}$ | 57 | 3.5x${10}^{-7}$ | 56 | 7.07x${10}^{-3}$ |
| *ITK* | 8.33x${10}^{-3}$ | 13 | 1.05x${10}^{-1}$ | 28 | 9.31x${10}^{-6}$ | 29 | 5.94x${10}^{-3}$ | 10 | 2.31x${10}^{-1}$ | 20 | 2.66x${10}^{-5}$ | 21 | 8.36x${10}^{-3}$ | 12 | 1.75x${10}^{-1}$ | 28 | 9.31x${10}^{-6}$ | 28 | 2.41x${10}^{-3}$ |
| *DDR1* | 1.22x${10}^{-3}$ | 5 | 5.2x${10}^{-3}$ | 6 | 9.8x${10}^{-8}$ | 6 | 1.66x${10}^{-6}$ | 4 | 6.62x${10}^{-3}$ | 4 | 1.11x${10}^{-6}$ | 4 | 3.87x${10}^{-6}$ | 5 | 5.2x${10}^{-3}$ | 6 | 9.8x${10}^{-8}$ | 6 | 1.66x${10}^{-6}$ |
| *TNXB* | 7.14x${10}^{-3}$ | 6 | 5.54x${10}^{-3}$ | 17 | 7.03x${10}^{-3}$ | 21 | 3.81x${10}^{-2}$ | 1 | -x${10}^{NA}$ | 9 | 6.91x${10}^{-3}$ | 11 | 7.65x${10}^{-3}$ | 3 | 1.26x${10}^{-1}$ | 15 | 2.84x${10}^{-3}$ | 17 | 5.45x${10}^{-3}$ |
| *ARMC2* | 5x${10}^{-2}$ | 55 | 9.82x${10}^{-11}$ | 63 | 3.91x${10}^{-5}$ | 68 | 7.16x${10}^{-6}$ | 29 | 4.27x${10}^{-7}$ | 39 | 1.45x${10}^{-3}$ | 44 | 2.19x${10}^{-3}$ | 39 | 4.71x${10}^{-7}$ | 58 | 1.33x${10}^{-5}$ | 62 | 1.94x${10}^{-5}$ |
| *LOC153910* | 2.5x${10}^{-2}$ | 28 | 3.06x${10}^{-10}$ | 44 | 3.15x${10}^{-4}$ | 45 | 2.15x${10}^{-1}$ | 19 | 2.83x${10}^{-9}$ | 28 | 8.85x${10}^{-5}$ | 30 | 1.17x${10}^{-1}$ | 22 | 3.12x${10}^{-10}$ | 41 | 1.82x${10}^{-5}$ | 43 | 1.56x${10}^{-1}$ |
| *PTCH1* | 5x${10}^{-2}$ | 22 | 6.15x${10}^{-14}$ | 57 | 5.21x${10}^{-4}$ | 54 | 1.66x${10}^{-1}$ | 6 | 1.64x${10}^{-2}$ | 27 | 1.71x${10}^{-2}$ | 26 | 2.99x${10}^{-1}$ | 13 | 3.07x${10}^{-3}$ | 41 | 3.87x${10}^{-3}$ | 40 | 2.34x${10}^{-1}$ |
| *CDC123* | 1.67x${10}^{-2}$ | 10 | 7.91x${10}^{-4}$ | 18 | 2.86x${10}^{-3}$ | 23 | 4.78x${10}^{-1}$ | 7 | 3.03x${10}^{-3}$ | 11 | 6.57x${10}^{-3}$ | 14 | 5.82x${10}^{-1}$ | 10 | 7.91x${10}^{-4}$ | 18 | 2.86x${10}^{-3}$ | 22 | 4.78x${10}^{-1}$ |
| *NUDT5* | 1.67x${10}^{-2}$ | 12 | 5.96x${10}^{-3}$ | 22 | 1.44x${10}^{-2}$ | 26 | 3.29x${10}^{-3}$ | 7 | 8.47x${10}^{-4}$ | 11 | 1.36x${10}^{-3}$ | 13 | 2.72x${10}^{-3}$ | 11 | 9.6x${10}^{-4}$ | 18 | 2.02x${10}^{-4}$ | 20 | 6.13x${10}^{-4}$ |
| *C10orf11* | 5x${10}^{-2}$ | 221 | 5.53x${10}^{-32}$ | 370 | 7.13x${10}^{-10}$ | 389 | 9.73x${10}^{-17}$ | 102 | 3.95x${10}^{-18}$ | 163 | 7.56x${10}^{-9}$ | 176 | 9.54x${10}^{-6}$ | 149 | 3.45x${10}^{-24}$ | 275 | 1.48x${10}^{-12}$ | 292 | 2.85x${10}^{-15}$ |
| *HAL* | 8.3x${10}^{-3}$ | 7 | 2.84x${10}^{-2}$ | 17 | 2.01x${10}^{-3}$ | 19 | 1.08x${10}^{-2}$ | 5 | 4.8x${10}^{-2}$ | 10 | 2.89x${10}^{-3}$ | 11 | 3.39x${10}^{-3}$ | 7 | 2.84x${10}^{-2}$ | 16 | 1.62x${10}^{-3}$ | 18 | 7.76x${10}^{-3}$ |
| *NTN4* | 8.3x${10}^{-3}$ | 43 | 1.44x${10}^{-6}$ | 57 | 1.39x${10}^{-2}$ | 62 | 1.36x${10}^{-6}$ | 21 | 1.73x${10}^{-8}$ | 31 | 8.06x${10}^{-3}$ | 35 | 3.06x${10}^{-9}$ | 28 | 1.6x${10}^{-7}$ | 47 | 5.11x${10}^{-3}$ | 50 | 1.64x${10}^{-8}$ |
| *THSD4* | 5x${10}^{-2}$ | 150 | 2.1x${10}^{-39}$ | 293 | 3.37x${10}^{-6}$ | 340 | 4.89x${10}^{-3}$ | 87 | 3.68x${10}^{-24}$ | 144 | 6.88x${10}^{-10}$ | 157 | 2.41x${10}^{-6}$ | 111 | 9.78x${10}^{-30}$ | 224 | 4.51x${10}^{-13}$ | 243 | 1.97x${10}^{-5}$ |
| *CNGB1* | 1x${10}^{-2}$ | 9 | 1.88x${10}^{-3}$ | 25 | 2.14x${10}^{-3}$ | 25 | 4.16x${10}^{-2}$ | 7 | 1.14x${10}^{-2}$ | 18 | 5.04x${10}^{-4}$ | 18 | 1.36x${10}^{-2}$ | 7 | 1.14x${10}^{-2}$ | 21 | 2.47x${10}^{-4}$ | 21 | 1.55x${10}^{-2}$ |
| *MMP15* | 1x${10}^{-2}$ | 5 | 2.78x${10}^{-3}$ | 5 | 7x${10}^{-8}$ | 6 | 3.89x${10}^{-6}$ | 4 | 3.5x${10}^{-3}$ | 3 | 3.25x${10}^{-7}$ | 4 | 8.63x${10}^{-6}$ | 5 | 2.78x${10}^{-3}$ | 4 | 2.63x${10}^{-7}$ | 5 | 7x${10}^{-6}$ |

1. Exon based

| **Locus** | **Threshold** | **All variants** | | | | | | **Independent variants** | | | | | | **Independent variants and variants**  **not in UK10K+1000G** | | | | | |
| --- | --- | --- | --- | --- | --- | --- | --- | --- | --- | --- | --- | --- | --- | --- | --- | --- | --- | --- | --- |
|  |  | **vipR** | | **SNVer** | | **Syzygy** | | **vipR** | | **SNVer** | | **Syzygy** | | **vipR** | | **SNVer** | | **Syzygy** | |
|  |  | **N** | **P** | **N** | **P** | **N** | **P** | **N** | **P** | **N** | **P** | **N** | **P** | **N** | **P** | **N** | **P** | **N** | **P** |
| *HDAC4* | 5x${10}^{-2}$ | 1 | - | 4 | 3.66x${10}^{-11}$ | 4 | 2.11x${10}^{-8}$ | 1 | - | 4 | 3.66x${10}^{-11}$ | 4 | 2.11x${10}^{-8}$ | 1 | - | 4 | 3.66x${10}^{-11}$ | 4 | 2.11x${10}^{-8}$ |
| *NPNT* | 1.25x${10}^{-2}$ | 7 | 3.38x${10}^{-6}$ | 10 | 6.76x${10}^{-5}$ | 12 | 5.36x${10}^{-4}$ | 3 | 1.81x${10}^{-3}$ | 6 | 2.88x${10}^{-3}$ | 5 | 1.75x${10}^{-1}$ | 3 | 1.81x${10}^{-3}$ | 8 | 2.84x${10}^{-3}$ | 7 | 1.96x${10}^{-1}$ |
