# Supplementary material for: Targeted Sequencing of Lung Function Loci in Chronic Obstructive Pulmonary Disease Cases and Controls
Source: PLoS One. 2017 Jan 23;12(1):e0170222. doi: 10.1371/journal.pone.0170222 (PMC5256917; doi:10.1371/journal.pone.0170222)
Supplement: S8 Table — “GWAS gene” is the gene reported in the lung function GWAS [9] for each region. P-values that reach a Bonferroni corrected threshold as defined in the methods section are highlighted in bold. a) Burden test results in stage 2b) C-alpha test results in stage 2iv) Sliding windowv) Gene basedvi) Exon based (DOCX) [file pone.0170222.s011.docx]

S8 Table Collapsing methods stage 2 results

“GWAS gene” is the gene reported in the lung function GWAS [[5](#_ENREF_5)] for each region. P-values that reach a Bonferroni corrected threshold as defined in the methods section are highlighted in bold.

1. Burden test results in stage 2

| **Locus** | **GWAS gene** | **Number of variants** | **P-value** |
| --- | --- | --- | --- |
| chr3:168984786-168987786 | *MECOM* | 2 | 4.15x${10}^{-1}$ |
| *FLJ20184* (chr4:106473776-106552837) | *GSTCD* | 46 | 3.84x${10}^{-1}$ |
| chr4:145278600-145281600 | *HHIP* | 2 | 8.83x${10}^{-1}$ |
| *ITK* (chr5: 156607906-156682109) | *ADAM19* | 23 | 7.58x${10}^{-1}$ |
| *GPR126* (chr6:142623055-142767403) | *GPR126* | 82 | 8.7x${10}^{-1}$ |

1. C-alpha test results in stage 2
2. Sliding window

| **Locus** | **GWAS gene** | **Number of variants** | **Number of alternative allele counts in cases and controls** | **P-value** |
| --- | --- | --- | --- | --- |
| chr1:218531175-218534175 | *TGFB2* | 3 | 421 | 8.13x${10}^{-1}$ |
| chr2:218807794-218810794 | *TNS1* | 2 | 244 | 7.57x${10}^{-1}$ |
| chr2:239973116-239976116 | *HDAC4* | 2 | 147 | 7.3x${10}^{-1}$ |
| chr2:240325616-240328616 | *HDAC4* | 3 | 401 | 6.57x${10}^{-1}$ |
| chr3:168984786-168987786 | *MECOM* | 2 | 252 | 8.25x${10}^{-1}$ |
| chr3:169238286-169241286 | *MECOM* | 2 | 573 | **2.94x**$\boldsymbol{10}^{\boldsymbol{-3}}$ |
| chr3:169310286-169313286 | *MECOM* | 4 | 604 | 8.35x${10}^{-1}$ |
| chr3:169311786-169314786 | *MECOM* | 2 | 238 | 6.73x${10}^{-1}$ |
| chr3:169340286-169343286 | *MECOM* | 3 | 665 | 8.87x${10}^{-1}$ |
| chr3:169341786-169344786 | *MECOM* | 3 | 590 | 8.71x${10}^{-1}$ |
| chr3:169371786-169374786 | *MECOM* | 5 | 908 | 5.26x${10}^{-1}$ |
| chr3:169373286-169376286 | *MECOM* | 4 | 607 | 1.99x${10}^{-1}$ |
| chr3:25464333-25467333 | *RARB* | 4 | 466 | 4.48x${10}^{-1}$ |
| chr3:25510833-25513833 | *RARB* | 6 | 1155 | 8.04x${10}^{-1}$ |
| chr3:25512333-25515333 | *RARB* | 4 | 878 | 7.54x${10}^{-1}$ |
| chr3:25632333-25635333 | *RARB* | 4 | 494 | 1.51x${10}^{-1}$ |
| chr3:25633833-25636833 | *RARB* | 3 | 363 | 5.32x${10}^{-2}$ |
| chr4:145269600-145272600 | *HHIP* | 2 | 158 | 7.54x${10}^{-1}$ |
| chr4:145278600-145281600 | *HHIP* | 2 | 210 | 7.82x${10}^{-1}$ |
| chr4:145293600-145296600 | *HHIP* | 2 | 167 | 4.16x${10}^{-2}$ |
| chr4:145341600-145344600 | *HHIP* | 2 | 299 | 4.18x${10}^{-1}$ |
| chr5:147829118-147832118 | *HTR5* | 3 | 276 | 5.75x${10}^{-1}$ |
| chr5:147830618-147833618 | *HTR6* | 4 | 597 | 7.83x${10}^{-1}$ |
| chr5:156912906-156915906 | *ADAM19* | 2 | 480 | 6.32x${10}^{-1}$ |
| chr9:98180197-98183197 | *PTCH1* | 6 | 565 | 4.81x${10}^{-1}$ |
| chr9:98181697-98184697 | *PTCH1* | 4 | 358 | 6.61x${10}^{-1}$ |
| chr10:12207674-12210674 | *CDC123* | 2 | 423 | 8.36x${10}^{-1}$ |
| chr12:57529676-57532676 | *LRP1* | 3 | 280 | 3.34x${10}^{-1}$ |
| chr12:96157082-96160082 | *CCDC38* | 4 | 856 | 8.65x${10}^{-1}$ |
| chr12:96158582-96161582 | *CCDC38* | 5 | 1115 | 8.85x${10}^{-1}$ |
| chr15:71704287-71707287 | *THSD4* | 2 | 205 | 7.5x${10}^{-1}$ |
| chr21:35646821-35649821 | *KCNE2* | 2 | 245 | 5.77x${10}^{-1}$ |

1. Gene based

| **Locus** | **GWAS gene** | **Number of variants** | **Number of alternative allele counts in cases and controls** | **P-value** |
| --- | --- | --- | --- | --- |
| *TGFB2* (chr1:218518675-218617961) | *TGFB2* | 65 | 7090 | 6.58x${10}^{-1}$ |
| *TNS1* (chr2:218664511-218808796) | *TNS1* | 84 | 13588 | 1.55x${10}^{-1}$ |
| *HDAC4* (chr2:239969863-240322643) | *HDAC4* | 247 | 34906 | 9.59x${10}^{-1}$ |
| *RARB* (chr3:25469833-25639422) | *RARB* | 76 | 9629 | 8.39x${10}^{-1}$ |
| *MECOM* (chr3:168801286-169381563) | *MECOM* | 450 | 69543 | 1 |
| *FAM13A* (chr4:89647105-89978323) | *FAM13A* | 123 | 13561 | 6.55x${10}^{-1}$ |
| *FLJ20184* (chr4:106473776-106552837) | *GSTCD* | 46 | 5725 | 6.25x${10}^{-1}$ |
| *HHIP* (chr4:145567147-145659881) | *HHIP* | 31 | 3882 | 7.94x${10}^{-1}$ |
| *ITK* (chr5:156607906-156682109) | *ADAM19* | 23 | 3253 | 5.33x${10}^{-1}$ |
| *DDR1* (chr6:30856464-30867933) | *NCR3* | 7 | 953 | 7.56x${10}^{-1}$ |
| *TNXB* (chr6:32008931-32077151) | *AGER* | 37 | 8086 | 6.08x${10}^{-2}$ |
| *ARMC2* (chr6:109169618-109295352) | *ARMC2* | 65 | 8782 | 9.21x${10}^{-1}$ |
| *LOC153910* (chr6:142847591-142958973) | *GPR126* | 86 | 12820 | 5.46x${10}^{-1}$ |
| *PTCH1* (chr9:98205263-98270831) | *PTCH1* | 52 | 6850 | 2.58x${10}^{-1}$ |
| *NUDT5* (chr10:12209572-12238143) | *CDC123* | 12 | 1894 | 9.24x${10}^{-1}$ |
| *CDC123* (chr10:12237960-12292589) | *CDC123* | 32 | 4619 | 9.4x${10}^{-1}$ |
| *C10orf11* (chr10:77542518-78317126) | *C10orf11* | 304 | 39634 | **4.03x**$\boldsymbol{10}^{\boldsymbol{-2}}$ |
| *NTN4* (chr12:96051582-96184536) | *CCDC38* | 88 | 14103 | 1 |
| *HAL* (chr12:96367141-96390071) | *CCDC38* | 14 | 1407 | 7.11x${10}^{-1}$ |
| *THSD4* (chr15:71433787-72075722) | *THSD4* | 291 | 38799 | 7.86x${10}^{-1}$ |
| *CNGB1* (chr16:57916243-58005020) | *MMP15* | 44 | 5877 | 9.61x${10}^{-1}$ |
| *MMP15* (chr16:58059281-58080804) | *MMP15* | 20 | 2098 | 3.92x${10}^{-1}$ |

1. Exon based

| **Locus** | **GWAS gene** | **Number of variants** | **Number of alternative allele counts in cases and controls** | **P-value** |
| --- | --- | --- | --- | --- |
| *HDAC4* (chr2:239969863-240322643*)* | *HDAC4* | 7 | 996 | 7.43x${10}^{-1}$ |
| *NPNT* (chr4:106816596-106892828*)* | *GSTCD* | 9 | 1400 | 5.25x${10}^{-2}$ |
