# Supplementary material for: Targeted Sequencing of Lung Function Loci in Chronic Obstructive Pulmonary Disease Cases and Controls
Source: PLoS One. 2017 Jan 23;12(1):e0170222. doi: 10.1371/journal.pone.0170222 (PMC5256917; doi:10.1371/journal.pone.0170222)
Supplement: S1 Supplementary References — (DOCX) [file pone.0170222.s012.docx]

# Supplementary References

1. Sherry ST, Ward MH, Kholodov M, Baker J, Phan L, Smigielski EM, et al. dbSNP: the NCBI database of genetic variation. Nucleic acids research. 2001;29(1):308-11. PubMed PMID: 11125122; PubMed Central PMCID: PMC29783.

2. 1000 Genomes Project Consortium, Abecasis GR, Auton A, Brooks LD, DePristo MA, Durbin RM, et al. An integrated map of genetic variation from 1,092 human genomes. Nature. 2012;491(7422):56-65. Epub 2012/11/07. doi: 10.1038/nature11632. PubMed PMID: 23128226; PubMed Central PMCID: PMC3498066.

3. Mills RE, Luttig CT, Larkins CE, Beauchamp A, Tsui C, Pittard WS, et al. An initial map of insertion and deletion (INDEL) variation in the human genome. Genome research. 2006;16(9):1182-90. doi: 10.1101/gr.4565806. PubMed PMID: 16902084; PubMed Central PMCID: PMC1557762.

4. UK10K Consortium, Walter K, Min JL, Huang J, Crooks L, Memari Y, et al. The UK10K project identifies rare variants in health and disease. Nature. 2015;526(7571):82-90. Epub 2015/09/15. doi: 10.1038/nature14962. PubMed PMID: 26367797.

5. Soler Artigas M, Loth DW, Wain LV, Gharib SA, Obeidat M, Tang W, et al. Genome-wide association and large-scale follow up identifies 16 new loci influencing lung function. Nature genetics. 2011;43(11):1082-90. Epub 2011/09/29. doi: 10.1038/ng.941. PubMed PMID: 21946350; PubMed Central PMCID: PMC3267376.

6. Hancock DB, Eijgelsheim M, Wilk JB, Gharib SA, Loehr LR, Marciante KD, et al. Meta-analyses of genome-wide association studies identify multiple loci associated with pulmonary function. Nature genetics. 2010;42(1):45-52. PubMed PMID: 20010835.

7. Wilk JB, Chen TH, Gottlieb DJ, Walter RE, Nagle MW, Brandler BJ, et al. A genome-wide association study of pulmonary function measures in the Framingham Heart Study. PLoS Genet. 2009;5(3):e1000429. Epub 2009/03/21. doi: 10.1371/journal.pgen.1000429. PubMed PMID: 19300500.

8. Repapi E, Sayers I, Wain LV, Burton PR, Johnson T, Obeidat M, et al. Genome-wide association study identifies five loci associated with lung function. Nature genetics. 2010;42(1):36-44. PubMed PMID: 20010834.
